# Exploring the cellular and molecular differences between ovarian clear cell carcinoma and high-grade serous carcinoma using single-cell RNA sequencing and GEO gene expression signatures

**DOI:** 10.1186/s13578-023-01087-3

**Published:** 2023-07-31

**Authors:** Dan Guo, Sumei Zhang, Yike Gao, Jinghua Shi, Xiaoxi Wang, Zixin Zhang, Yaran Zhang, Yuming Wang, Kun Zhao, Mei Li, Anqi Wang, Pan Wang, Yanqin Gou, Miao Zhang, Meiyu Liu, Yuhan Zhang, Rui Chen, Jian Sun, Shu Wang, Xunyao Wu, Zhiyong Liang, Jie Chen, Jinghe Lang

**Affiliations:** 1 Clinical Biobank, Peking Union Medical College Hospital, Chinese Academy of Medical Sciences & Peking Union Medical College, Beijing, China; 2 Department of Medical Research Center, Peking Union Medical College Hospital, Chinese Academy of Medical Sciences & Peking Union Medical College, Beijing, China; 3 Department of Pathology, Molecular Pathology Research Center, Peking Union Medical College Hospital, Chinese Academy of Medical Science & Peking Union Medical College, Beijing, China; 4 Department of Obstetrics and Gynecology, Peking Union Medical College Hospital, Chinese Academy of Medical Sciences & Peking Union Medical College, Beijing, China; 5 National Clinical Research Center for Obstetric & Gynecologic Diseases, Beijing, China; 6 Department of Pathology, Affiliated Hospital of Hebei University, Baoding, China; 7 Department of Pathology, People’s Hospital of Ningxia Hui Autonomous Region, Yinchuan, China

**Keywords:** OCCC, HGSC, Single-cell RNA-seq, Bulk RNA-seq

## Abstract

The two most prevalent subtypes of epithelial ovarian carcinoma (EOC) are ovarian clear cell carcinoma (OCCC) and high-grade serous ovarian carcinoma (HGSC). Patients with OCCC have a poor prognosis than those with HGSC due to chemoresistance, implying the need for novel treatment target. In this study, we applied single-cell RNA sequencing (scRNA-seq) together with bulk RNA-seq data from the GEO (Gene Expression Omnibus) database (the GSE189553 dataset) to characterize and compare tumor heterogeneity and cell-level evolution between OCCC and HGSC samples. To begin, we found that the smaller proportion of an epithelial OCCC cell subset in the G2/M phase might explain OCCC chemoresistance. Second, we identified a possible pathogenic OCCC epithelial cell subcluster that overexpresses LEFTY1. Third, novel biomarkers separating OCCC from HGSC were discovered and subsequently validated on a wide scale using immunohistochemistry. Amine oxidase copper containing 1 (*AOC1*) was preferentially expressed in OCCC over HGSC, while S100 calcium-binding protein A2 (*S100A2*) was detected less frequently in OCCC than in HGSC. In addition, we discovered that metabolic pathways were enriched in the epithelial compartment of the OCCC samples. In vitro experiments verified that inhibition of oxidative phosphorylation or glycolysis pathways exerted direct antitumor effects on both OCCC and HGSC cells, while targeting glutamine metabolism or ferroptosis greatly attenuated chemosensitivity only in OCCC cells. Finally, to determine whether there were any variations in immune cell subsets between OCCC and HGSC, data from scRNA-seq and mass cytometry were pooled for analysis. In summary, our work provides the first holistic insights into the cellular and molecular distinctions between OCCC and HGSC and is a valuable source for discovering new targets to leverage in clinical treatments to improve the poor prognosis of patients with OCCC.

**Supplementary Information:**

The online version contains supplementary material available at 10.1186/s13578-023-01087-3.

## Introduction

Epithelial ovarian carcinoma (EOC) is the predominantly lethal gynecological cancer and ranks seventh among the most commonly diagnosed cancer in women worldwide [1]. High-grade serous ovarian cancer (HGSC) is the most common EOC and accounts for as many as 60% of EOC cases. Ovarian clear cell carcinoma (OCCC) is the second most common EOC, accounting for 5–11% of EOC cases [2, 3].

The demographic, morphological, and molecular features between OCCC and HGSC differ. OCCC is more prevalent in Asians than in people of other races and is closely related to endometriosis [4]. OCCC displays a tubulocystic architecture and clear cells, while HGSC is characterized by solid and papillary structures. OCCC is characterized by the overexpression of HNF1B [5, 6] and is typically ER- and PR-negative. However, most HGSC cells harbor TP53 and WT-1 mutations [7]. In addition, frequent ER expression and low PR expression increase the likelihood that HGSC is hormone-dependent. HGSC and estrogen or progesterone have been linked in a number of studies [8–10].

Surgical procedure with subsequent chemotherapy is the conventional treatment for OCCC and HGSC. However, OCCC leads to a worse prognosis than HSGC due to OCCC chemoresistance to conventional platinum [11]. Therefore, exploring the cellular and molecular differences between OCCC and HGSC will help in developing alternative therapies.

Single-cell RNA sequencing (scRNA-seq) has emerged as a powerful tool to explore intratumor heterogeneity and evolution at the cell level. It can be used to help interpret the pathogenic mechanism and offers the possibility of personalized treatment. In the present study, we applied scRNA-seq and performed a combined analysis with bulk RNA-seq data from the GEO database to systematically characterize the cellular and molecular differences between OCCC and HGSC (Figure S1). Our study offers a valuable resource for exploring new therapeutic targets of OCCC or both clinically.

## Results

### A higher proportion of LEFTY1^+^ and a lower proportion of the G2/M phase epithelial subset in OCCC compared with HGSC

We performed 3’ scRNA-seq with samples collected during surgery from five OCCC and five HGSC patients. Detailed clinical information was provided in Table S1. Representative images of H&E and IHC stained samples were presented in Figure S2A. After filtering low-quality cells and inferred doublets, we integrated all ten samples into a gene expression matrix. As a result, we obtained a total of 101,672 cells with 2000 genes on average in each cell. We performed unsupervised clustering analysis before performing a broad comparison of different cell types in the tumor tissues obtained from OCCC and HGSC patients. The cells were clustered into three main populations: epithelial, immune, and stromal cells (Figure S2B-C).

**Fig. 1:**
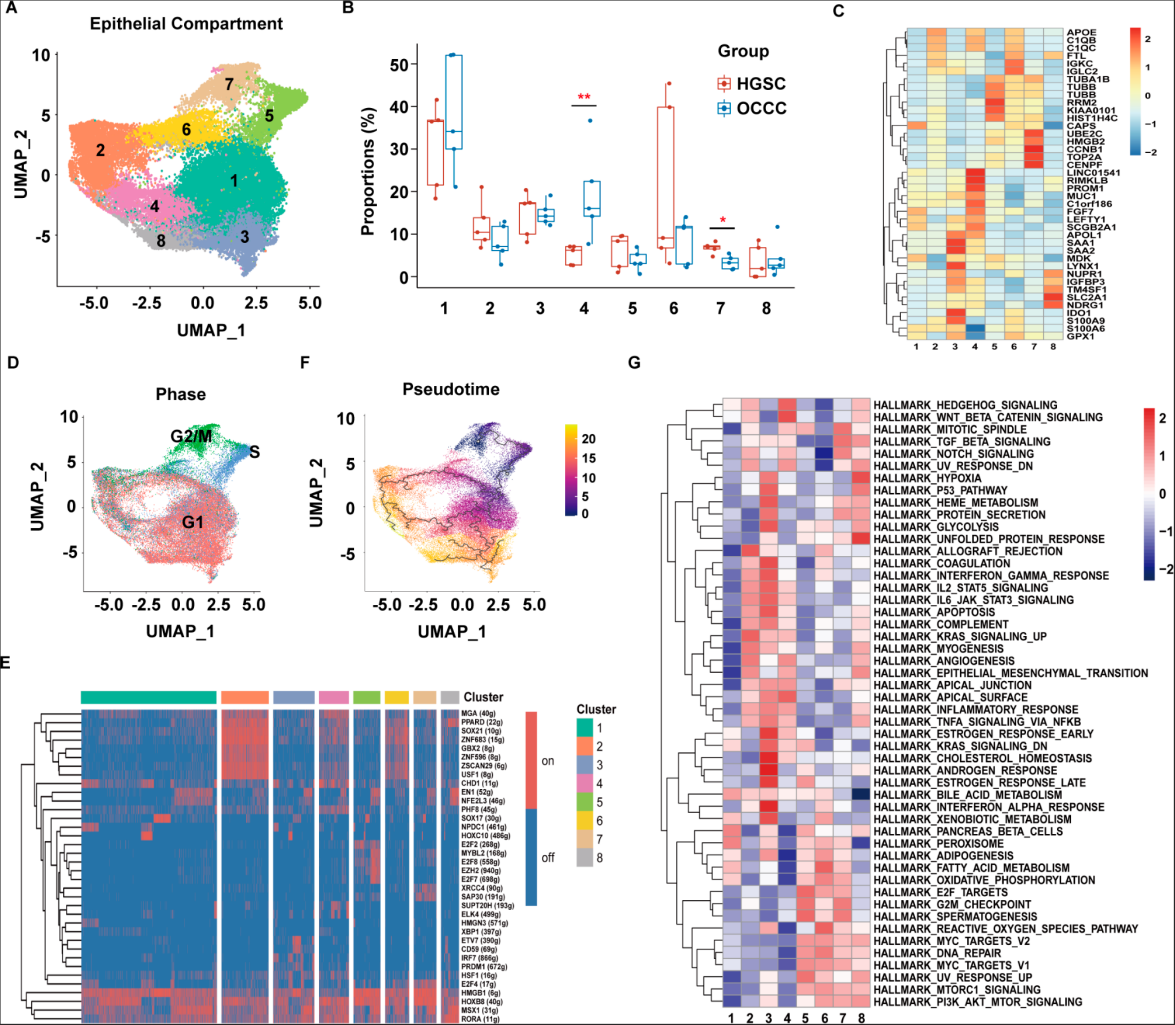
A higher proportion of LEFTY1 + epithelial subset cells and a lower proportion of epithelial subset cells in the G2/M phase were observed in OCCC samples than in HGSC samples. **(A)** UMAP plot displaying eight epithelial cell subpopulations. Each dot represents a single cell (n = 39,722). **(B)** The box plot shows the comparison of each epithelial cell percentage in the OCCC and HGSC groups. **(C)** Heatmap showing the average expression of the top 5 most highly expressed markers among epithelial cell subsets. **(D)** UMAP plot displaying the epithelial cell subsets in each cell cycle phase. **(E)** Heatmap showing regulon activity as analyzed by SCENIC. A “regulon” refers to the regulatory network of TFs and their target genes. “On” indicates active regulons; “Off” indicates inactive regulons. **(F)** Pseudotime reconstruction and development of epithelial cell subsets inferred from Monocle 3. **(G)** Pathway analysis of each epithelial cluster. *P* values were calculated by two-sided Wilcoxon test. *: *p* < 0.05, **: *p* < 0.01

We then performed an unsupervised sub-clustering analysis and identified eight epithelial subpopulations (Fig. 1A). The large-scale copy number variation (CNV) analysis of malignant cells showed fewer CNVs across the whole genome in all subsets of OCCC cells than in the HGSC cell subsets (Figure S3). The proportion analysis showed that more cells were classified into Cluster 4 and fewer cells were classified into Cluster 7 in OCCC compared with HGSC (Fig. 1B).

The identification of feature markers showed that Cluster 4 expressed *MUC1*, *FGF7*, and left-right determination factor 1 (*LEFTY1)*, and Cluster 7 expressed cell cycle-related genes (Fig. 1C). An analysis of the proportion of cells in each cell cycle phase reflected that Cluster 5 cells were arrested mostly in the S phase, while Cluster 7 cells were mainly arrested in the G2/M phase (Fig. 1D). Investigation into the regulon activity using SCENIC showed that the proliferation-related transcription factor *HMGB1* was activated in Cluster 5 and Cluster 7 cells but was inactive in Cluster 4 (Fig. 1E).

To explore the development of epithelial cell subsets, we performed a pseudotime trajectory analysis using Monocle 3 based on expression and transition profiles (Fig. 1F). Interestingly, we observed that Cluster 5 cells (S phase) bifurcated into two branches leading to other branches, namely, directly to Cluster 7 or Cluster 1, when in an intermediate state to other clusters (Fig. 1F). Pathway enrichment analyses of each epithelial subtype showed that Cluster 4 was enriched with “Hedgehog signaling”, “Wnt-beta catenin signaling”, “TGF-beta signaling”, “Notch signaling” and “angiogenesis” (Fig. 1G). Taken together, based on the lower proportion of OCCC cells in Cluster 7 and higher proportions of OCCC cells in Cluster 4 compared to the proportion of HGSC cells in these clusters, as described above, we speculated that Cluster 5 OCCC cells might show an increased capacity to develop into Cluster 4 cells but a decreased capacity to develop into Cluster 7 cells compared with the HGSC cells in these clusters.

### Identification and validation of distinct molecular markers in OCCC and HGSC cells discovered by combining scRNA-seq and bulk RNA-seq data from the GEO database

**Fig. 2:**
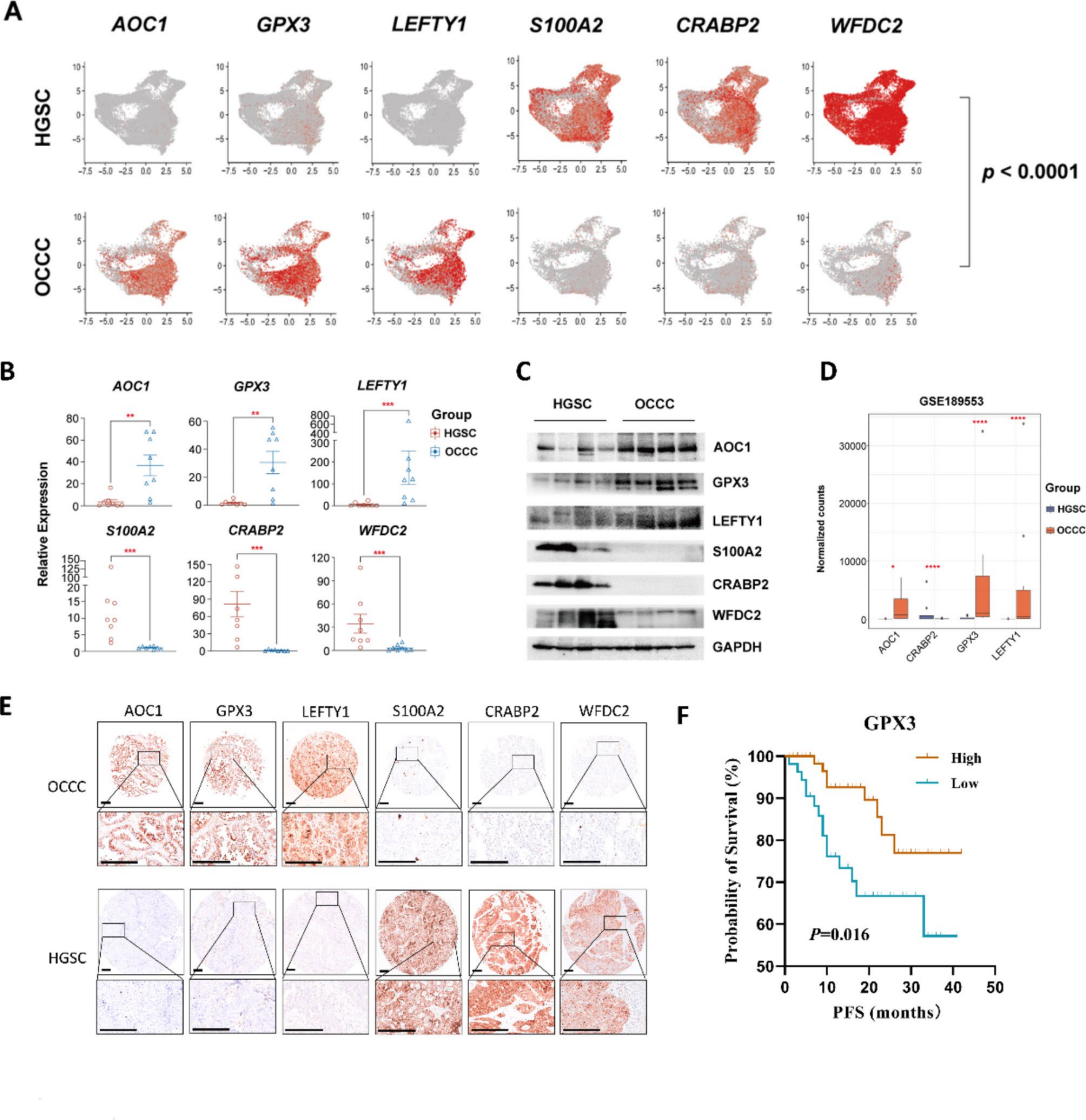
Detection and validation of new biomarkers in OCCC patients. **(A)** Comparisons of *AOC1*, *GPX3*, *LEFTY1*, *S100A, CRABP2* and *WFDC2* expression levels as shown in a UMAP plot. Each dot represents a single cell. **(B)***AOC1*, *GPX3*, *LEFTY1*, *S100A, CRABP2* and *WFDC2* expression levels in OCCC (n = 8) and HGSC (n = 8) patients, as determined by real-time PCR. Each dot represents a single individual. **(C)** Western blot results of AOC1, GPX3, LEFTY1, S100A, CRABP2 and WFDC2 expression in OCCC (n = 4) and HGSC (n = 4) patients. **(D)** Comparisons of *AOC1*, *GPX3*, *LEFTY1* and *CRABP2* expression in the GSE189553 dataset. **(E)** Representative images of AOC1, GPX3, LEFTY1, S100A, CRABP2 and WFDC2 in the TMA of OCCC and HGSC samples after immunohistochemical staining. The scale bar is 250 μm. **(F)** The progression-free survival (PFS) analysis results based on GPX3 level in OCCC patients. *P* values were calculated by two-sided Wilcoxon test. *: *p* < 0.05, **: *p* < 0.01, ***: *p* < 0.001, ****: *p* < 0.0001

A differentially expressed gene (DEG) analysis of epithelial cell subpopulations between OCCC and HGSC patients showed that the expression levels of amine oxidase copper containing 1 (*AOC1*), left-right determination factor 1 (*LEFTY1*), and glutathione peroxidase 3 (*GPX3*) were significantly higher in OCCC samples than in HGSC samples, while the opposite was true for S100 calcium-binding protein A2 (*S100A2*), WAP four-disulfide core domain 2 (*WFDC2*), and cellular retinoic acid-binding protein 2 (*CRABP2*) (Fig. 2A). Further, RT‒PCR and immunoblotting supported these observations (Fig. 2B-C, Figure S4). The DEG analysis with the GSE189553 dataset validated that OCCC samples showed higher expression of *AOC1*, *LEFTY1*, and *GPX3*, and lower expression of *CRABP2* compared to HGSC samples (Fig. 2D).

We constructed two tissue microarrays (TMAs) consisting of 128 OCCC samples and 81 HGSC samples. Representative images were illustrated in Fig. 2E and Figure S5. The IHC validation cohort confirmed that AOC1, LEFTY1, and GPX3 were more expressed in the OCCC samples, while WFDC2, CRABP2, and S100A2 were more expressed in the HGSC samples (Table 1). We analyzed the association of clinical features and IHC results with progression-free survival (PFS). The high expression of GPX3 was significantly associated with a longer PFS than low expression in OCCC samples (Fig. 2F). As shown in Table S2, in the univariate analysis, early stage, no residual tumor after primary cytoreductive surgery and high expression of GPX3 were related to a better prognosis for OCCC patients (*p* = 0.010, 0.013, 0.021, respectively). Early stage (*p* = 0.014) and expression of GPX3 (*p* = 0.029) were still correlated with a longer PFS in OCCC patients in the multivariate analysis. For the HGSC group, residual tumor and chemoresistance were significantly related to prognostic data (*p* = 0.015 and < 0.001, respectively), while chemoresistance was a susceptibility factor for recurrence (*p* = 0.001). Moreover, a significantly lower proportion of GPX3 expression was found in the platinum-resistant (PR) subgroup than in the platinum-sensitive (PS) subgroup of OCCC patients (Table 2).

**Table 1:** Comparison of immunohistochemical results with OCCC and HGSC samples

	OCCC (n = 128)	HGSC (n = 81)	*p* value
AOC1	80 (62.5%)	9 (11.1%)	< 0.001^a^
GPX3	71 (55.5%)	3 (3.7%)	< 0.001^a^
LEFTY1	119 (93.0%)	38 (46.9%)	< 0.001^a^
S100A2	4 (3.1%)	27 (33.3%)	< 0.001^a^
CRABP2	41 (32.0%)	74 (91.4%)	< 0.001^a^
WFDC2	32 (25%)	56 (69.1%)	< 0.001^a^

Values indicate the number of patient from which samples showed high immunohistochemical staining (percentages).

a: statistically significant.

**Table 2:** Association of identified markers with chemoresistance

	OCCC			HGSC		
	PR (n = 15)	PS (n = 113)	*p* value	PR (n = 2)	PS (n = 79)	*p* value
AOC1	6 (40.0%)	74 (65.5%)	0.055	0 (0)	9 (11.4%)	1.000
GPX3	4 (26.7%)	67 (59.3%)	0.017^a^	0 (0)	3 (3.8%)	1.000
LEFTY1	14 (93.3%)	105 (92.9%)	1.000	2 (100.0%)	36 (45.6%)	0.217
S100A2	1 (6.7%)	3 (2.7%)	0.396	1 (50.0%)	26 (32.9%)	1.000
CRABP2	7 (46.7%)	34 (30.1%)	0.318	2 (100.0%)	72 (91.1%)	1.000
WFDC2	1 (6.7%)	31 (27.2%)	0.158	2 (100.0%)	54 (68.4%)	1.000

Values indicate the number of patients for whom samples showed high immunohistochemical staining (percentages)

PR: platinum-resistant; PS: platinum-sensitive

a: statistically significant

### Metabolism pathways were activated and might be promising targets for OCCC treatment

**Fig. 3:**
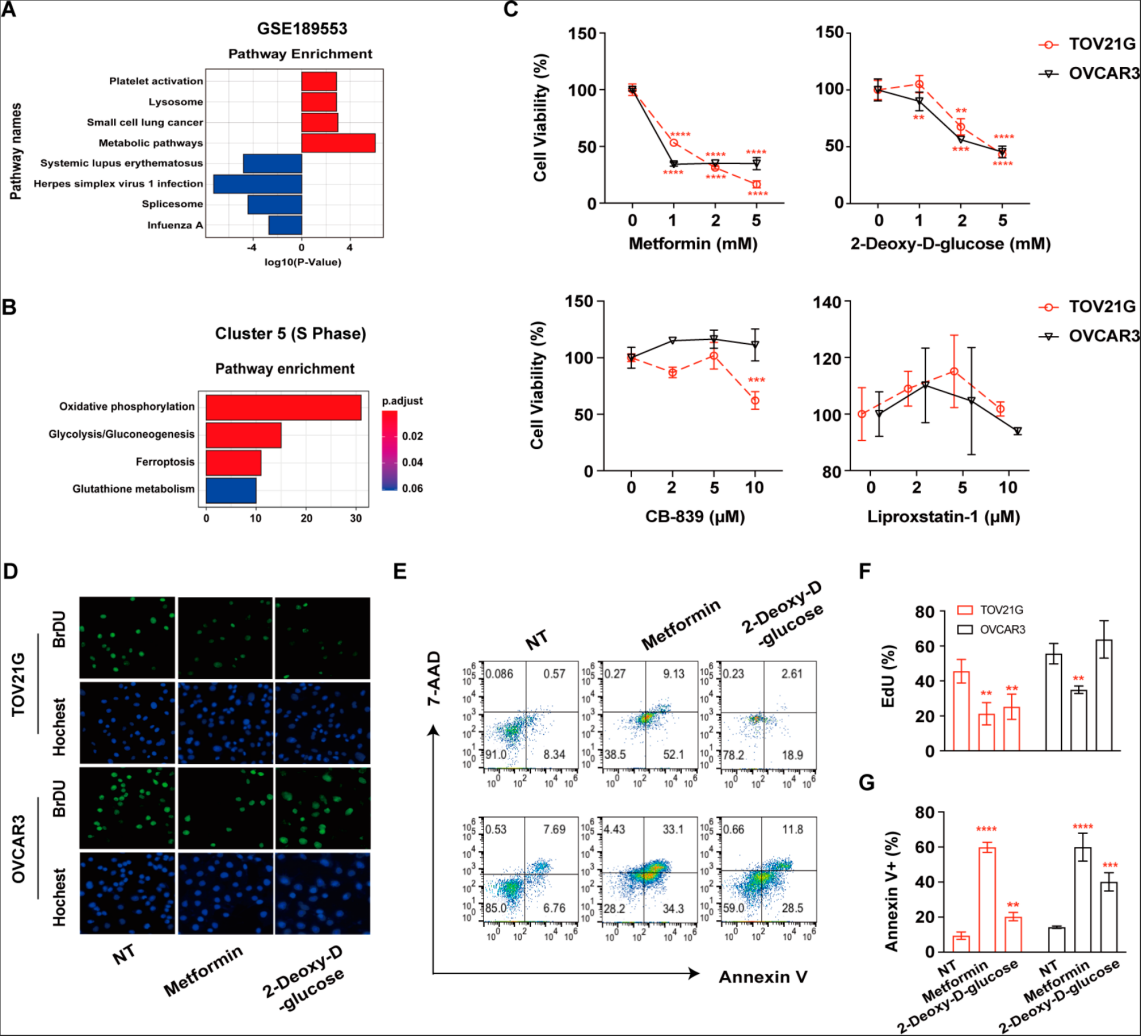
Glucose metabolism pathways are activated in OCCC cells and might be promising targets for both OCCC and HGSC treatments as indicated by in vitro experiments. **(A)** Bar plots showing the differentially expressed genes (DEGs) in the GSE189553 dataset with enriched Kyoto Encyclopedia of Genes and Genomes (KEGG) pathways. Red: OCCC; blue: HGSC. **(B)** Bar plots showing the differentially expressed gene (DEG)-enriched Kyoto Encyclopedia of Genes and Genomes (KEGG) pathways in Cluster 5 consisting of OCCC and HGSC patient cells. **(C)** TOV21G and OVCAR3 cells were treated with the indicated concentrations of CB-839, liproxstatin-1, metformin, and 2-deoxy-D-glucose, and cell viability was measured by CCK8 assay 72 h after treatment. **(D)** TOV21G/OVCAR3 cells were treated with metformin (5 mM) and 2-deoxy-D-glucose (5 mM) for 48 h. Representative images of EdU-positive cells by immunofluorescence. **(E)** TOV21G/OVCAR3 cells were treated with metformin (5 mM) and 2-deoxy-D-glucose (5 mM) for 72 h. Representative images of flow cytometry are presented. **(F)** Summary of the percentage of EdU-positive cells. **(G)** The percentage of Annexin V-positive cells was determined by flow cytometry. NT: no treatment. All assays were carried out in triplicate, and the data are presented as the mean ± S.D. * *p* < 0.05, ** *p* < 0.01, *** *p* < 0.001, **** *p* < 0.0001

Signaling pathway enrichment of differentially expressed genes (DEGs) in the GSE189553 dataset showed that OCCC cells were enriched with “metabolic pathways” (Fig. 3A). Accordingly, signaling pathway enrichment with DEGs in Cluster 5 (dividing cells, S phase) between the OCCC and HGSC populations as distinguished with single-cell RNA seq data revealed that OCCC patient samples were enriched with genes related to “oxidative phosphorylation”, “glutathione metabolism”, “glycolysis/gluconeogenesis” and “ferroptosis” pathways (Fig. 3B).

We further treated the TOV21G cell line (an OCCC cell line) and OVCAR3 cell line (an HGSC cell line) with CB-839, liproxstatin-1, metformin, and 2-deoxy-D-glucose to explore the potential roles of glutamine metabolism, ferroptosis, oxidative phosphorylation, and the glycolysis pathway in OCCC and HGSC cells. The viability of both cell lines was inhibited by metformin or 2-deoxy-D-glucose treatment, which indicated aberrant glucose metabolism in both OCCC and HGSC cells (Fig. 3C). Cell proliferation via EdU assay and apoptosis via flow cytometry revealed that metformin inhibited cell proliferation and promoted cell apoptosis in TOV21G and OVCAR3 cells (Fig. 3D-F). 2-Deoxy-D-glucose functioned through different mechanisms to reduce cell viability. It induced anti-proliferation effects against TOV21G cells and promoted apoptosis in OVCAR3 (Fig. 3D-F).

**Fig. 4:**
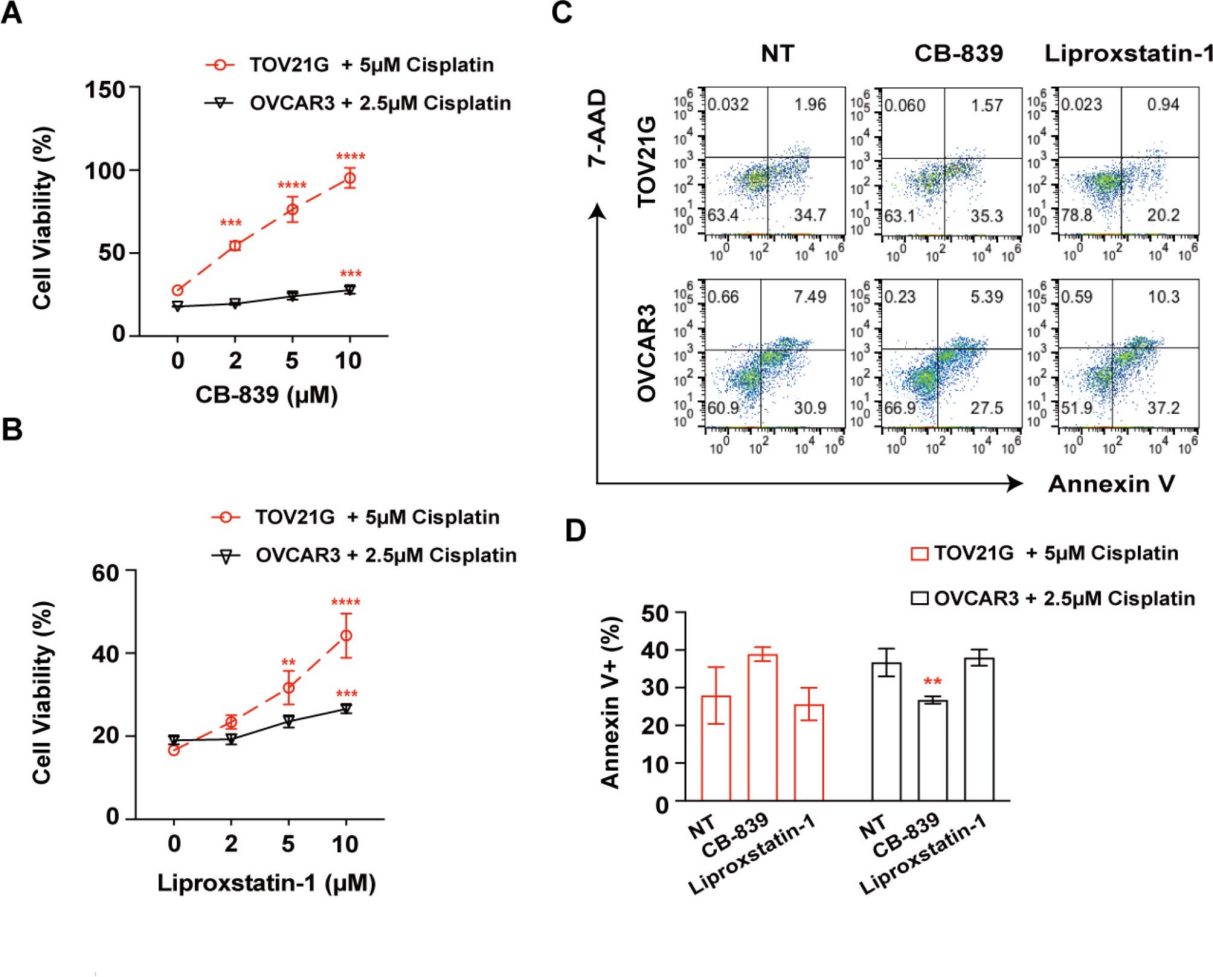
Inhibition of glutathione metabolism or ferroptosis reverses cisplatin-induced death of OCCC cells, as determined in vitro. **(A)** TOV21G or OVCAR3 cells were treated with the indicated concentrations of CB-839 with cisplatin. Cell viability was measured by CCK8 assay 72 h after treatment. **(B)** TOV21G or OVCAR3 cells were treated with the indicated concentrations of liproxstatin-1 with cisplatin. Cell viability was measured by CCK8 assay 72 h after treatment. **(C-D)** TOV21G/OVCAR3 cells were treated with CB-839 (10 𝛍M) and liproxstatin-1 (10 𝛍M) with cisplatin for 72 h. Representative images and summary of the percentage of Annexin V-positive cells as determined by flow cytometry NT: no treatment. All assays were carried out in triplicate, and the data are presented as the mean ± S.D. * *p* < 0.05, ** *p* < 0.01, *** *p* < 0.001, **** *p* < 0.0001

Interestingly, we found that the inhibition of the glutathione metabolism pathway or ferroptosis by CB-839 or lip-1 significantly increased the viability of the TOV21G cell line treated with cisplatin (Fig. 4A-B). Only a mild effect was observed in the OVCAR3 cell line (Fig. 4A-B). However, this inhibitory effect was not associated with reduced cisplatin-induced apoptosis (Fig. 4C-D). These results indicated that metformin and 2-deoxy-D-glucose show good potential as therapeutic drugs. Targeting glutamine metabolism or ferroptosis greatly attenuated chemosensitivity only in OCCC cells.

### Heterogeneity of immune cells in patients with OCCC or HGSC

**Fig. 5:**
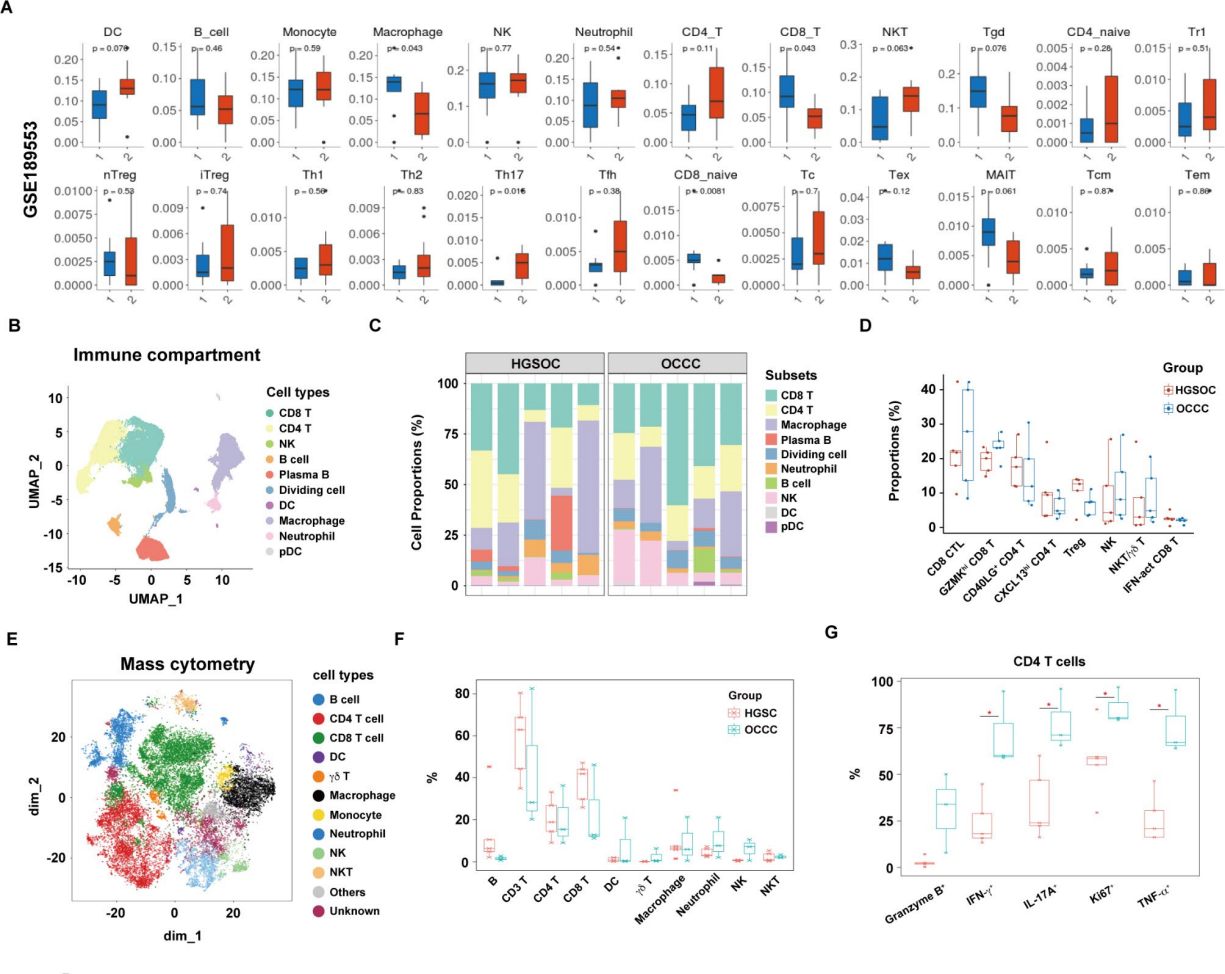
Comparison of immune cell heterogeneity in OCCC and HGSC samples. **(A**) Comparisons of immune cell frequency and T-cell subset frequency between OCCC [1] and HGSC [2] in the GSE189553 dataset as determined by ImmunCellAI analysis. DC: Dendritic cell; B: B cell; NK: natural killer cell; NKT: natural killer T cell; nTreg: natural regulatory T cell; iTreg: induced regulatory T cell; Th: T helper; Tfh: follicular helper T; MAIT: mucosal-associated invariant T; Tcm: central memory T; Tem: effector memory T. **(B)** UMAP plot displaying ten immune subpopulations (n = 48,213). **(C)** The distribution and proportion of ten immune subsets in each sample of the HGSC and OCCC groups from scRNA-seq data. **(D)** Box plots showing a comparison of the percentage of each T/NK cluster between the HGSC and OCCC groups. **(E)** t-SNE plot showing 12 clusters of TILs from mass cytometry data. Each dot represents a single cell (n = 48,213). **(F)** Box plots showing the comparison of the percentages of each TIL cluster between the HGSC and OCCC groups. **(G)** Box plots showing the comparison of different marker expression levels in CD4 T cells in the HGSC and OCCC groups. Each dot represents a single cell. *P* values were calculated by two-sided Wilcoxon test. * *p* < 0.05

Next, we compared the extent of tumor-infiltrating immune cells between OCCC and HGSC patients. We used ImmunCellAI to estimate and compare the immune cell abundance in the GSE189553 dataset. We found higher percentages of macrophages and CD8^+^ T cells in OCCC patients than in HGSC patients (Fig. 5A). In addition, an analysis of T-cell subsets revealed lower percentages of Th17 cells and naïve CD8^+^ T (CD8_Naive) cells in OCCC patients than in HGSC patients (Fig. 5A).

We produced subsets of immune cells from scRNA-seq data and reidentified ten immune cell populations, including CD8 T cells, CD4 T cells, NK cells, B cells, plasma B cells, dividing cells, DCs, macrophages, neutrophils and pDCs, on the basis of canonical marker levels, (Fig. 5B, Figure S6A). We found that T cells were predominantly enriched in tumor-infiltrating immune cells of HGSC and OCCC patients; however, the proportions of immune cell subsets were similar in OCCC samples compared to those in HGSC samples (Fig. 5C). We further examined the cellular subtypes in the T/NK subsets and identified eight subpopulations: CD8 CTLs, GZMK^hi^ CD8 T cells, IFN-act CD8 T cells, NK cells, NKT/γδ T cells, GD40LG^+^ CD4 T cells, CXCL13^hi^ CD4 T cells, and Tregs (Figure S6B-C). No significant differences were found in the proportions of these eight T/NK subpopulations between OCCC and HGSC samples (Fig. 5D).

We performed mass cytometry and identified nine immune cell populations, namely, in CD45^+^ (tumor-infiltrating lymphocytes) TILs, including B cells, CD4^+^ T cells, CD8^+^ T cells, DCs, γδ T cells, macrophages/monocytes, neutrophils, NK cells, and NKT cells using canonical marker level measurements (Fig. 5E, Figure S6D). Consistent with our scRNA-seq analysis results, T cells (CD4^+^ and CD8^+^ T) accounted for a large proportion of CD45^+^ TILs, and no significant difference was observed in the percentages of B cells, CD4 T cells, CD8 T cells, DCs, NK cells, macrophages and neutrophils (Fig. 5F). However, the levels of a proliferation marker (Ki-67) and inflammatory cytokine markers, including IFN-γ, IL-17 A, and TNF-α were significantly higher in CD4^+^ T cells but not CD8^+^ T cells in OCCC than in HGSC (Fig. 5G, Figure S6E).

## Discussion

Our study is the first to explore the cellular and molecular differences in primary tumor tissues between OCCC and HGSC using single-cell RNA sequencing and GEO gene expression signatures. We partially explained the chemoresistance of OCCC cells via a cell cycle analysis. We also identified a possible pathogenic epithelial subcluster with overexpressed LEFTY1 in OCCC samples. New biomarkers distinguishing OCCC samples from HGSC samples were identified, among which AOC1 and S100A2 were the first to be reported. Additionally, we showed that metabolic pathways were activated in OCCC cells, which indicates that they might be promising candidates for new therapeutic strategies. In addition, we characterized and compared the immune cellular profiles of tumor-infiltrating lymphocytes between OCCC and HGSC.

In the past two decades, RNA sequencing has become a ubiquitous tool used in molecular biology, and RNA sequencing results have had significant implications on the choices of specific molecular biomarkers and clinical approaches [12, 13]. Several studies that have applied scRNA-seq data to HGSC studies have suggested possible antitumor targets [14, 15], components of the tumor environment [16, 17], and chemotherapy resistance mechanisms [18]. However, the results of scRNA-seq analysis with OCCC samples have not been reported to date.

Cell cycle phase and SCENIC analyses in this study partially explained the chemoresistance mechanism underlying OCCC oncogenesis. Recent studies discovered that cisplatin caused cell death mainly by preventing RNA transcription [19] and induced cells to arrest in the G2 phase instead of the S phase [20]. The lower proportion of G2/M phase tumor cells that we found in OCCC samples might contribute to its higher platinum resistance than that of HGSC.

In this study, the dividing epithelial cell subset (Cluster 5) was more likely to develop into the *LEFTY1*^+^ subset (Cluster 4) in the OCCC group than in the HGSC group. The *LEFTY1*^+^ subset probably plays an important role in the pathogenesis of OCCC. LEFTY1 is a novel member of the TGF-beta superfamily and modulates the epithelial-mesenchymal transition and cancer stem cell properties in the context of OCCC [21, 22]. Other cancer-related pathways enriched in Cluster 4 cells, such as “Hedgehog signaling”, “Wnt-beta catenin signaling” and “Notch signaling”, had been reported in some studies on HGSC [23–25], but they had been rarely studied in the OCCC context. Further studies are needed to investigate whether inhibiting these signaling pathways may be an effective clinical therapy for OCCC.

In addition, fewer intensive CNVs were observed in OCCC cells than in HGSC cells. In a pancancer analysis, high CNV was associated with significantly worsened overall survival [26]. Late-stage tumors carry distinct features [27]; for example, CNV burden accumulates to a higher level in the deeper lesions of squamous esophageal carcinoma [28]. The higher CNV expression in HGSC cells may be a result of the late stages of HGSC patients compared with OCCC patients from which the samples were taken.

New diagnostic biomarkers were identified via the combined analysis of single-cell RNA sequencing and GEO gene expression data. Neither AOC1 nor S100A2 had been reported to distinguish OCCC samples from HGSC samples. AOC1, a copper-containing amine oxidase, plays different roles in different carcinomas. In gastric cancer and hepatocellular carcinoma, *AOC1* functions as an oncogene, either by activating the AKT signaling pathway and EMT [29] or by regulating the IL-6/JAK/STAT3 pathway [30]. However, AOC1 promotes ferroptosis and inhibits prostate cancer progression [31]. In our study, AOC1 was a promising feature marker of OCCC cells. Additionally, platinum-sensitive patients were more likely to overexpress AOC1 than platinum-resistant patients, with a *p* value close to being statistically significant (*p* = 0.055). S100A2, a member of the largest subfamily of calcium-binding EF-hand type proteins, has been found to be related to poor prognosis in patients with ovarian cancer [32]. Its expression is significantly higher in the advanced stage than in the early stage of an EOC [33]. In our study, the expression of S100A2 was detected more frequently in HGSC than in OCCC samples. S100A2 may be used to differentiate OCCC from HGSC samples.

Consistent with published studies, LEFTY1 and GPX3 were also identified in this study as potential OCCC molecular biomarkers. A previous study applied shotgun proteomics and identified LEFTY1 as a specific OCCC molecular marker, showing that this protein exerted an antitumor effect by reducing cell proliferation and promoting cisplatin-induced apoptosis [21]. Higher expression of GPX3 was discovered and found to be related to a better prognosis and chemosensitivity in patients with OCCC in our findings, which was in line with a previous study [34]. Additionally, we found a lower proportion of primary OCCC tumor tissues of OCCC expressing WFDC2 and CRABP2 compared than was found in HGSC tissues. WAP four-disulfide core domain 2 (WFDC2/HE4) is an EOC clinical biomarker [35–38], and cellular retinoic acid-binding protein 2 (CRABP2) is upregulated in ovarian cancer and contributes to tumor growth and tumor cell migration and invasion [39–41].

We found that inhibiting glucose metabolism by metformin or 2-deoxy-D-glucose effectively decreased the viability of OCCC and HGSC cell lines. Metformin is a well-characterized medication used for type 2 diabetes, and increasing evidence has highlighted its potential use as an affordable, well-tolerated, and effective agent anticancer and antiaging therapy [42–44]. Metformin has also been demonstrated to be a novel treatment option for ovarian cancer by either preventing tumor growth or increasing chemotherapy sensitivity [45–50]. A recent Phase II clinical trial evaluating metformin therapy suggested a better-than-expected overall survival for patients with ovarian cancer [51]. Our work first suggests an antitumor effect of metformin on OCCC patients because it suppresses tumor growth and promotes tumor cell apoptosis. Therefore, metformin can also be explored as a promising candidate for OCCC clinical treatment.

OCCC is often associated with endometriosis and is highly linked to high oxidative stress. Quenching reactive oxygen species (ROS) is expected to improve patient outcomes [52]. Glutathione is the most abundant cellular antioxidant that can neutralize ROS. Ferroptosis is an iron-dependent cell death associated with drug resistance in many cancers [53–55]. In endometriosis-related ovarian cancer, such as OCCC, repeated bleeding caused by endometriosis contributes to the accumulation of iron, which stimulates the Fenton reaction, produces ROS, and further induces ferroptosis [56]. Elevated intracellular iron levels are closely associated with ovarian cancer, and ferroptosis-inhibiting genes have been found to be related to ovarian cancer progression and chemoresistance [57–61]. Since our data suggest that inhibiting glutathione metabolism, which might lead to an increased ROS level, or inhibiting ROS-induced ferroptosis can greatly attenuated chemosensitivity in OCCC, it will be interesting to investigate whether and how ROS participate in OCCC chemoresistance.

Immune cells are important components in the tumor environment and exert an impact on treatment efficiency and prognosis [62–65]. Different immune cells participate in immune responses to tumors through different pathways [66, 67]. The scRNA-seq data combined with mass cytometry data in our study showed that T cells (CD4^+^ and CD8^+^ T) constitute a large proportion of CD45^+^ TILs in OCCC samples. The proportions of immune cell subsets did not differ. However, the results were limited by a low cell count and sequencing depth. Further study of the immune environment of OCCC and HGSC is needed.

There are, however, a number of restrictions on this study. To validate the results, we only performed TMA and in vitro cell line experiments. Further functional and mechanistic experiments are needed, in particular, murine in vivo or organoid models, to confirm our findings. Additionally, as was mentioned in the CNV section, selection bias might have occurred because to differences in the stages of the OCCC and HGSC patients.

In summary, our work provides holistic insights into the cellular and molecular differences between OCCC and HGSC samples. Specifically, we identified characteristic biomarkers, specific cell types, and metabolically related pathways involved in the tumorigenesis and drug resistance of OCCC in patients. Our study serves as a valuable resource for use in discovering new targets that improve the poor prognosis of OCCC patients.

## Materials and methods

### Patients

All patients underwent surgical resection at Peking Union Medical College Hospital. None of the patients had received therapy before surgery. Mixed carcinoma was ruled out in selected participants. In addition, patients with double primary carcinomas, namely, with both ovarian and uterine tumors, were excluded. The diagnosis was confirmed by 3 experienced pathologists, who evaluated tumors according to the 2020 World Health Organization classification [68], and staging was performed using The NCCN Guidelines for Ovarian Cancer (Version 3.2022) [69]. Clinical data, such as residual tumor after surgery, were gathered using the criteria of previous investigations [70, 71]. Therapeutic information included chemotherapy and and other therapies were also documented [70]. Chemoresistance was outlined as progression, persistent disease while receiving maintenance therapy, or full remission followed by relapse within six months of finishing platinum-based chemotherapy [65, 69].

### Single-cell suspension preparation

Single-cell preparation for scRNA-seq and mass cytometry validation was performed according to a previous study [16]. In brief, tumors were cut into approximately 1 mm^3^ pieces and digested in Hank’s balanced salt solution (HBSS) containing 1.5 mg/ml collagenase IV (Sigma‒Aldrich), 1 mg/ml hyaluronidase (Sigma‒Aldrich) and 500 µg/ml DNase I (GoldBio) while rotating at 200 rpm and 37 °C for 30 min. Cell suspensions were subsequently passed through a 70-µm cell strainer (BD, Biosciences), followed by centrifugation at 400 × g for 10 min. Red blood cells were lysed with RBC lysis buffer (Miltenyi Biotec, 130-094-183) for 2 min on ice, and dead cells were removed with a dead cell removal kit (Miltenyi Biotec, 130-090-101) following the manufacturer’s instructions.

### Library construction and single-cell sequencing

Library construction was conducted using a Chromium Next GEM Single Cell 3’ GEM Library & Gel Bead Kit v3.1 (10×Genomics, USA), and sequencing was performed with an Illumina NovaSeq 6000 (Novogene, Beijing, China).

### RNA-seq data processing

Raw sequencing data generated with the 10×Genomics platform were processed following the standard Chromium Cell Ranger pipeline (version 4.0.0) against the GRCh38 human reference genome. We filtered data and created Individual Seurat objects converted from single-cell counts from all samples using the Seurat analysis package in R (v4.1.0) according to a procedure described in a previous study [72]. We then merged all samples and performed a canonical correlation analysis (CCA) to reduce dimensionality and remove the batch effect. We then performed standard cell clustering using the *ScaleData* function. We used the *RunPCA* function to calculate the principal component analysis (PCA) dimensions and the *FindNeighbors* and *FindCluster* functions for unsupervised clustering of the data. Finally, we used the *RunUMAP* function for cell visualization.

### Cell subclustering analysis

Epithelial cells, immune cells (T cells, NK cells, myeloid cells, plasma B cells, B cells, and plasmacytoid dendritic cells (pDCs)), and stromal cells were extracted on the basis of integrated data for further subclustering. A similar process of clustering was performed for each major cell type to obtain cell subtypes. Doublet clusters were removed following the following criteria: [1] epithelial cells based on CD3 expression (calculated as the mean expression of *CD3D*, *CD3E*, and *CD3G*) > 0.1; [2] stromal cells based on *CD3D*, *C1QA*, *EPCAM*, and *CD14* expression.

### Differentially expressed genes and pathway enrichment

To identify differentially expressed genes (DEGs), we used the FindMarkers function with two-sided unpaired Wilcoxon tests, and *p* values were adjusted following the Benjamin & Hochberg protocol. DEGs were filtered with a minimum log_2_(fold change) of 0 and a maximum adjusted *p* value of 0.05. GO and KEGG pathway enrichment analyses were performed based on the DEGs using the R package ClusterProfiler (v4.0.5) [73].

### Trajectory analysis

A trajectory analysis of the epithelial cells was performed using Seurat and Monocle 3 R package software (v1.0.0) designed by Cao et al. [74]. In brief, we used the *GetAssayData* function to fetch the raw expression matrix, created the CellDataSet, and used the preprocess_cds function to normalize and preprocess data. The cluster_cells and learn_graph functions were used for the trajectory inference analysis, and the results were visualized using the UMAP function.

### CNV and SCENIC analysis

To identify malignant cells, we used InferCNV software (v1.2.0) to estimate the number of CNVs in each region [75]. The CNV was calculated based on the expression level for each cell with a cutoff of 0.1. T cells were used as the reference. We used the R package pySCENIC (v0.10.0) to analyze the enrichment of transcriptome factors in each epithelial subtype [76]. The activity of each regulon was evaluated using AUCell.

### Real-time PCR

Total RNA was extracted using TRIzol reagent (Thermo Fisher Scientific) and reverse transcribed into cDNA using PrimeScript™ RT Master Mix (TAKARA, Japan). Real-time PCR was performed using Fast SYBR Green Master Mix (Thermo Fisher Scientific) and a QuantStudio™ 7 Flex Real-Time PCR System (Thermo Fisher Scientific). Gene expression was normalized to GAPDH. Tables S3 and S4 provide detailed information on the primers used.

### Western blotting

Cells were lysed with RIPA lysis buffer (AOqing Biotechnology, Beijing, China) containing ProtLytic Protease Inhibitor Cocktail (New Cell & Molecular Biotech), and the protein concentration was determined with a BCA assay kit (Pierce Biotechnology, USA). The cell lysate was fractionated in NuPAGE™ 4 to 12% gels (Thermo Fisher Scientific) and transferred onto polyvinylidene difluoride (PVDF) membranes (Millipore, USA). The membranes were blocked and incubated with primary antibodies, including antibodies against LEFTY1, AOC1, GPX3, CRABP2, WFDC2, and S100A2, and then, the commensurate secondary antibody. The bands were visualized with enhanced chemiluminescence following the manufacturer’s instructions (Peirce).

### Gene set enrichment (GSE) analysis

We obtained profiling of gene expressions in primary ovarian cancer specimens (OCCC, n = 11, HGSC, n = 8) in the GSE189553 dataset from the GEO database (http://www.ncbi.nlm.nih.gov/geo/). DEGs between OCCC specimens and HGSC specimens were identified using the limma package (v3.50.3).

### Estimating the abundance of immune cell types via Immune cell abundance identifier (ImmunCellAI)

To estimate the abundance of 24 immune cell types, log2-transformed expression data from RNA-Seq results were compiled and uploaded to the web server for ImmunCellAI (http://bioinfo.life.hust.edu.cn/web/ImmuCellAI/) analysis as previously described [77].

### Immunohistochemical staining and interpretation

The IHC validation cohort consisted of patients diagnosed with OCCC and enrolled via consecutive sampling. The patients underwent surgical resection between January 2019 and May 2022 at Peking Union Medical College Hospital with enough archived tissue for immunohistochemical testing. Finally, 128 patients with OCCC and 81 patients with HGSC were included. The clinical information was listed in Table S5. Two tissue microarrays were constructed with formalin-fixed, paraffin-embedded tissues. IHC staining was performed with DAKO Autostainer Link 48 and measured according to the standard as reported in our previous studies [78, 79]. The primary antibodies were listed in Table S6.

The location of expressed protein and the tissue control in immunohistochemical staining were also listed in Table S7 [80–85]. Composite scores (range from 0 to 12) were calculated based on the intensity and percentage of stained cells [81, 86]. Cases with composite scores of 4 points or more were termed high expression, while the others were labeled low expression.

### Cell culture

The TOV21G cell line was purchased from ATCC (Manassas, VA, USA) and cultured in MCDB105 medium (Zhong Qiao Xin Zhou Biotechnology, Shanghai, China). The OVCAR3 cell line was purchased from the National Collection of Authenticated Cell Cultures (Shanghai, China) and cultured in RPMI-1640 medium (Zhong Qiao Xin Zhou Biotechnology, Shanghai, China) supplemented with 10% FBS (Gibco, USA) and 1% penicillin/streptomycin (Invitrogen, USA). Both cell lines were maintained in an incubator at 37 °C with 5% CO_2_.

### CCK8 assay

Cells were seeded at a density of 5000 cells per well in 96-well plates (Corning Life Sciences, USA), grown overnight, and exposed to different concentrations of cisplatin (HY-17,394, MedChemExpress) and treated with different inhibitors, including 2-deoxy-D-glucose (HY-13,966, MedChemExpress), metformin (PHR1084, Merck), CB-839 (HY-12,248, MedChemExpress), liproxstatin-1 (HY-12,726, MedChemExpress) and ferrostatin-1 (HY-100,579, MedChemExpress), for 24 h, 48 h, 72 h, followed by 4 h of incubation with 10 µl of CCK8 reagent (Dojindo, Shanghai, China). The absorbance at 450 nm was measured using a microplate reader (Bio–Rad, USA).

### Apoptosis rate determined by flow cytometry

Cells were seeded at a density of 5 × 10^5^ cells per well in a 6-well plate, grown overnight, and treated with cisplatin combined with different inhibitors as described above. The cell apoptosis rate was assessed with a FITC Annexin V Apoptosis Detection Kit (BD, Biosciences) following the manufacturer’s instructions. The data were obtained with an Attune NxT flow cytometer (Invitrogen).

### EdU assay

Cells were seeded at a density of 2 × 10^5^ cells per well in a 12-well plate, grown overnight, and treated with cisplatin combined with different inhibitors as described above. Cell proliferation was determined using a Click-iT® EdU Imaging Kit (C10337, Invitrogen) according to the manufacturer’s protocol. The nucleus was stained with 1 × Hoechst 33,342 solution (5 µg/mL). Images of five randomly selected areas were taken with a fluorescence microscope (Zeiss, Germany).

### Statistical analysis

GraphPad Prism software Version 8.0 (GraphPad, San Diego, CA) and SPSS Version 24.0 (SPSS Inc., Chicago, IL, USA) were used for statistical analyses and graphic presentations. The data are shown as the mean ± standard deviation (SD). Differences among multiple groups were compared by one-way ANOVA or Kruskal‒Wallis test with Bonferroni post hoc test based on a normal distribution and homogeneity of variance. Pearson’s chi-squared test and Fisher’s exact test were performed to evaluate binary variables, such as immunohistochemical results or application of chemotherapy. Univariate and multivariable analyses based on progression-free survival (PFS) were performed using Cox regression. The proportional hazards assumption was evaluated by analyzing the statistical significance of interactions between exposure and the follow-up time. A *P* value < 0.05 was considered statistically significant.

## Electronic supplementary material

Below is the link to the electronic supplementary material.

Figures and Tables

## Data Availability

The accession number for the raw sequencing data has been deposited in the Genome Sequence Archive for humans (GSA-Human) under accession number HRA002536. To comply with the “Guidance of the Ministry of Science and Technology (MOST) for the Review and Approval of Human Genetic Resources”, we are required to deposit the genomic data of Chinese people under controlled access at the GSA in Beijing Institute of Genomics Data Center. To access the raw data under accession number HRA002536, please submit requests to the GSA-Human online page for this study (https://ngdc.cncb.ac.cn).
