# Supplementary material for: Exploring the cellular and molecular differences between ovarian clear cell carcinoma and high-grade serous carcinoma using single-cell RNA sequencing and GEO gene expression signatures
Source: Cell Biosci. 2023 Jul 31;13:139. doi: 10.1186/s13578-023-01087-3 (PMC10391916; doi:10.1186/s13578-023-01087-3)
Supplement: Supplementary file 1 — Figures and Tables [file 13578_2023_1087_MOESM1_ESM.docx]

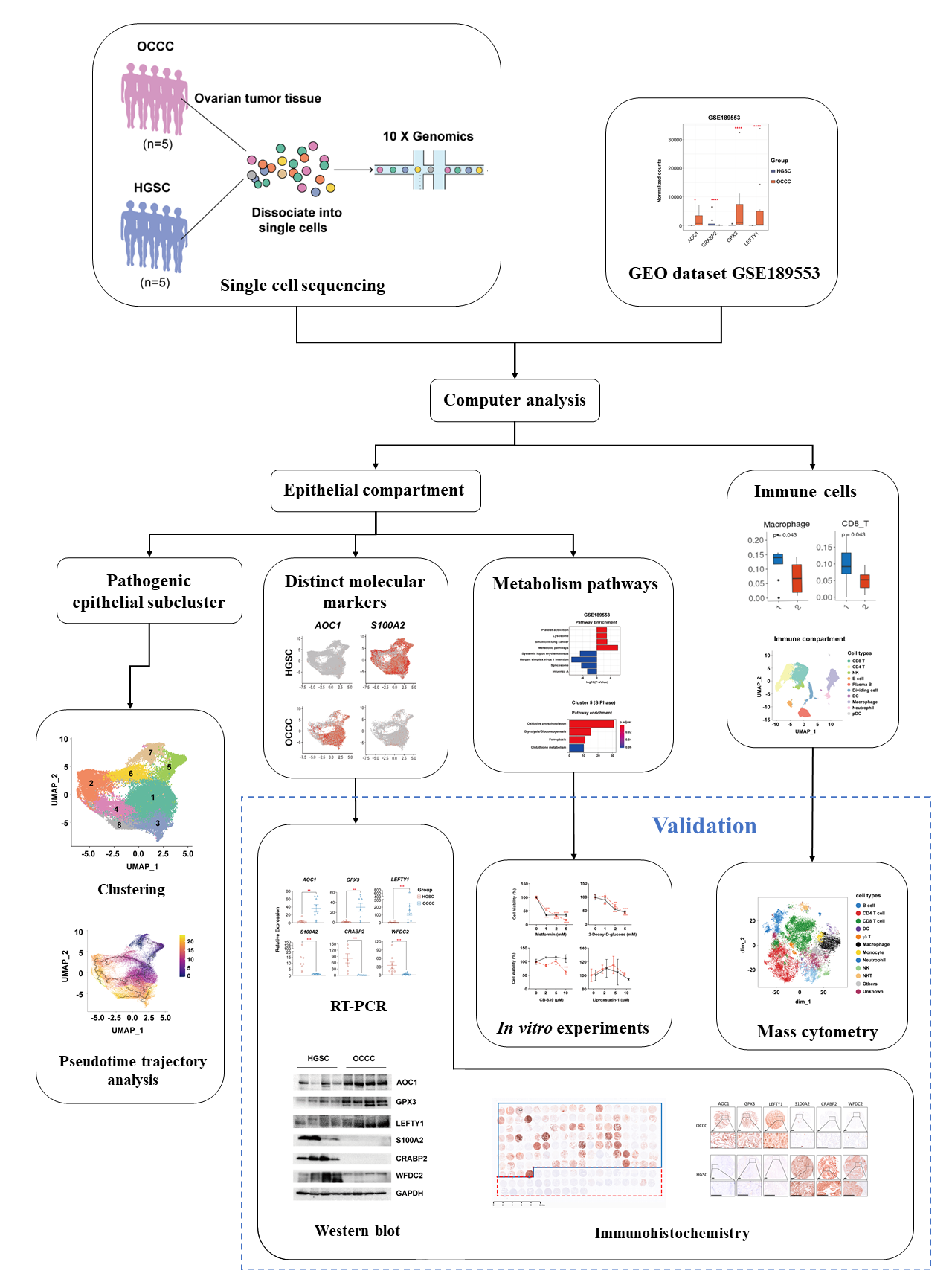


**Figure S1. The workflow of this study.**


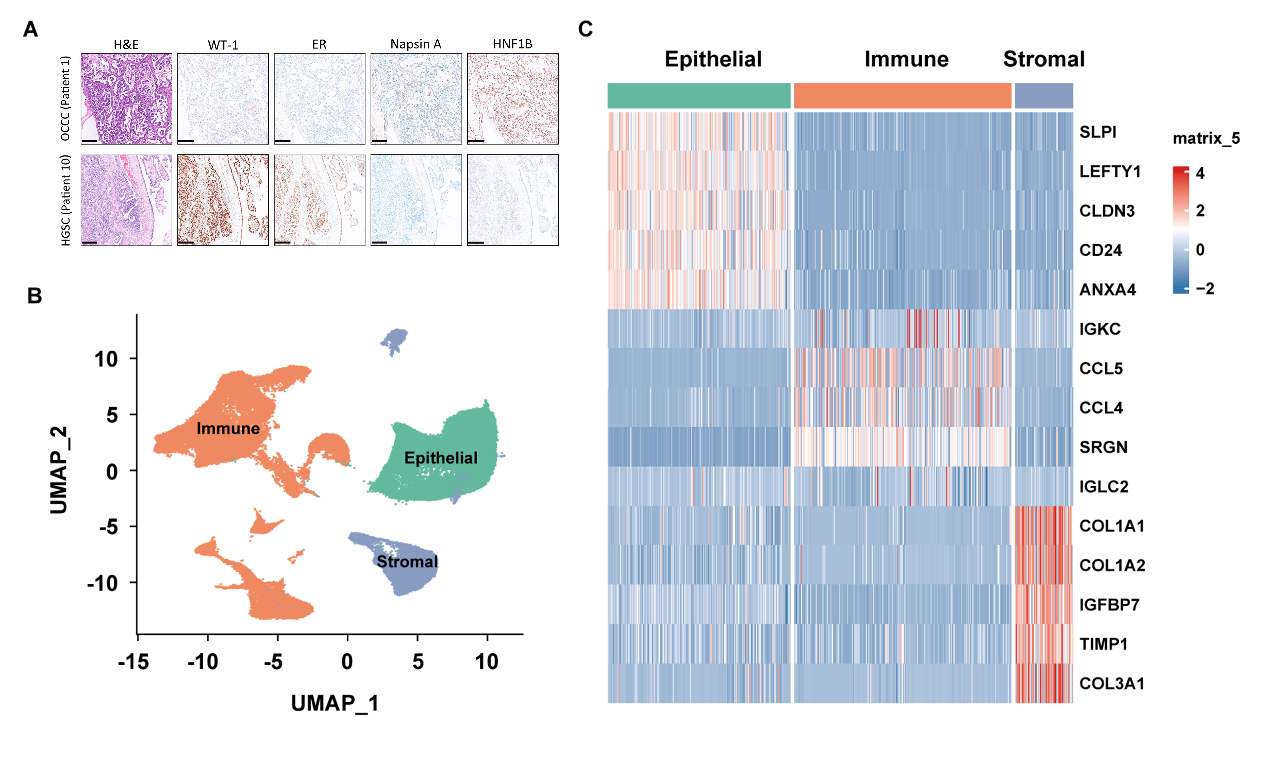


**Fig. S2. Landscape of single cell profile in tumors from OCCC and HGSC patients.**

**(A)** Hematoxylin and eosin (H&E) and immunohistochemically stained sections of OCCC and HGSC samples. WT-1, ER, Napsin A, and HNF1B were utilized in the differential diagnosis between OCCC and HGSC samples. The scale bar is 250 μm. **(B)** UMAP plot showing three major clusters of 101,672 cells. **(C)** Heatmap showing the average expression of signature genes for each of the three subclusters.


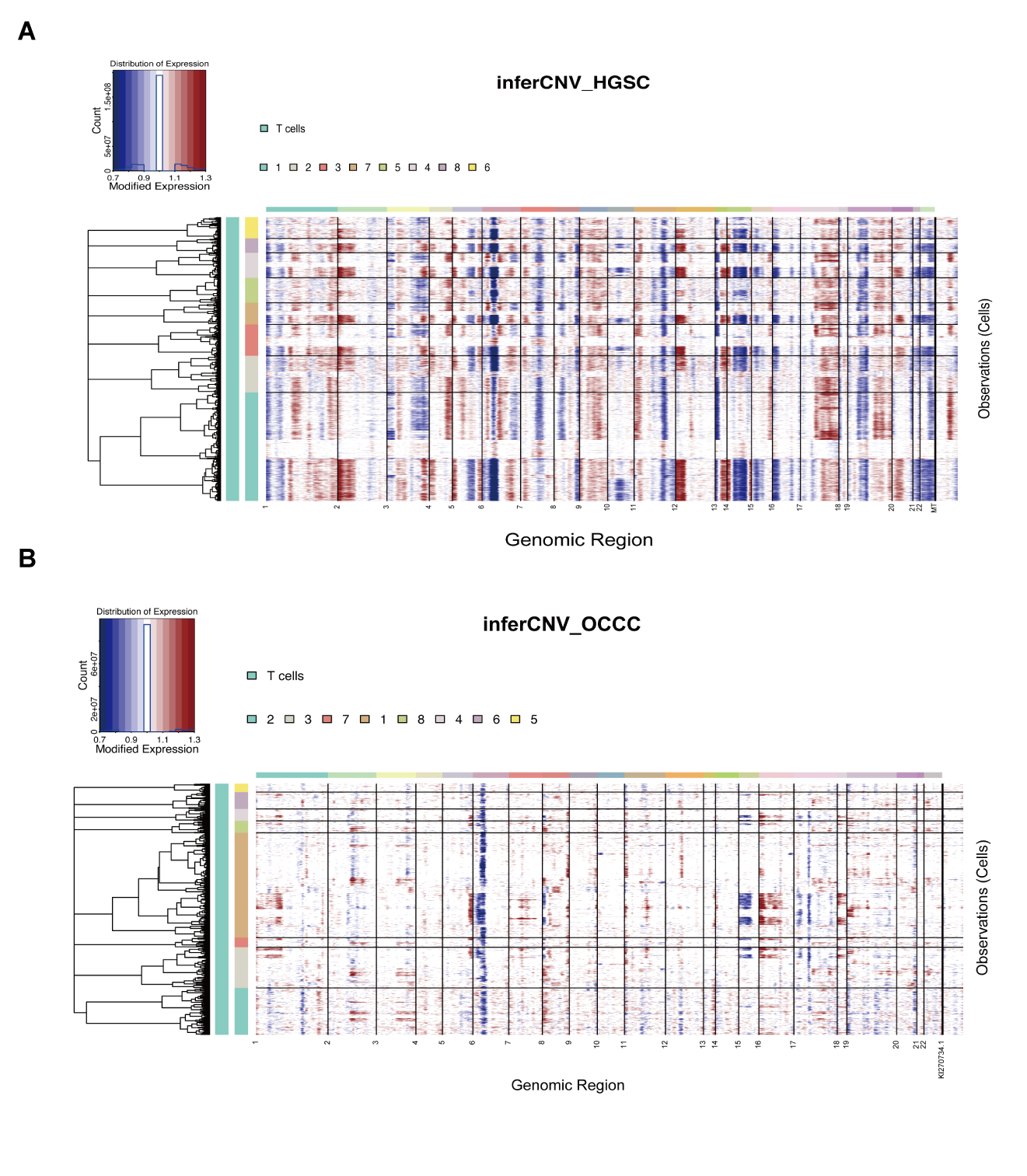
**Fig. S3. Large-scale CNV in the epithelium of OCCC and HGSC patients.**

Heatmap showing large-scale CNV for HGSC **(A)** and OCCC **(B)**, inferences based on the average expression of 100 genes surrounding each chromosomal position. Red: application; blue: deletion.


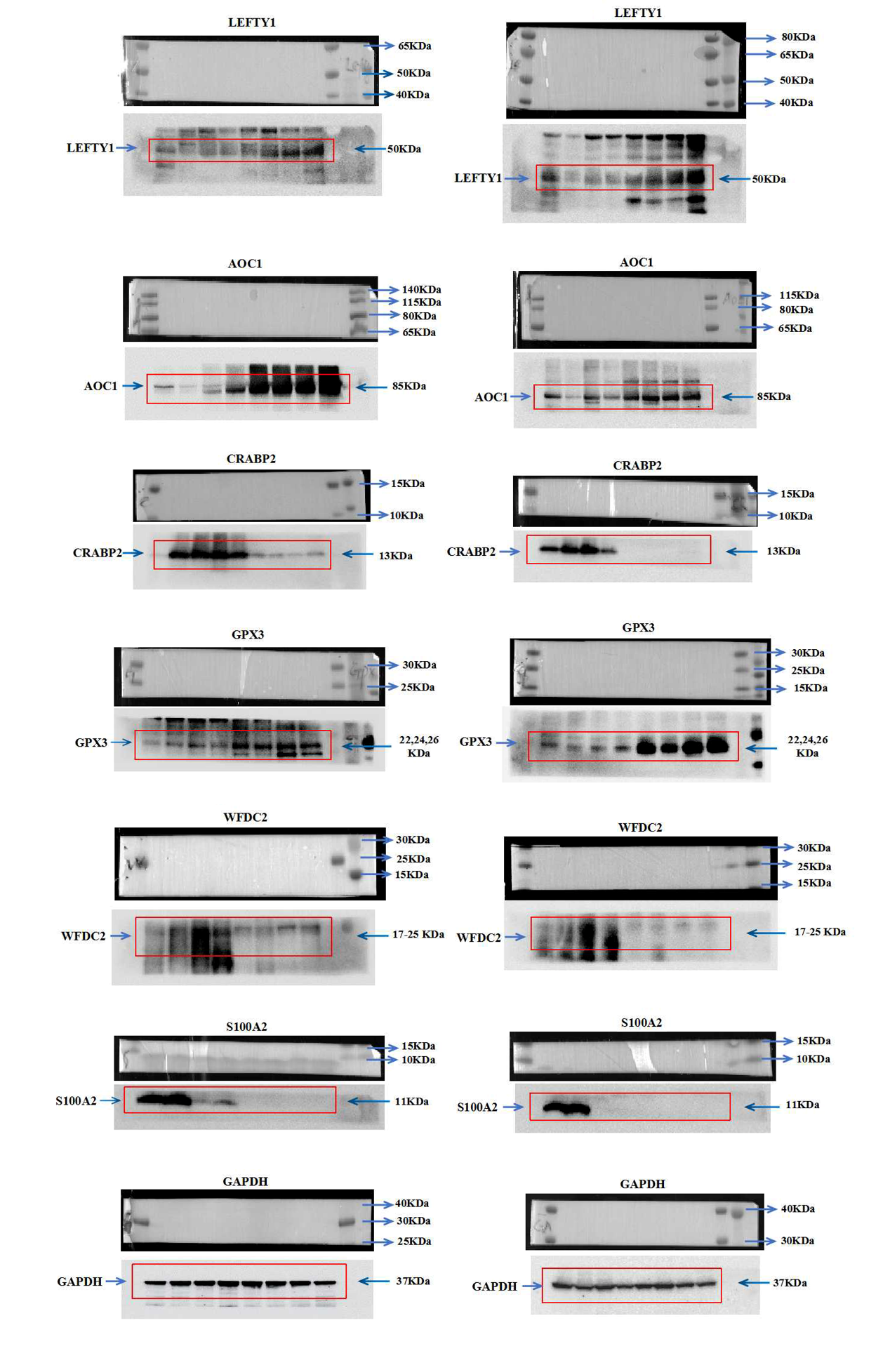


**Fig. S4. Western blot results in its original form.** The experienment was repeated twice.


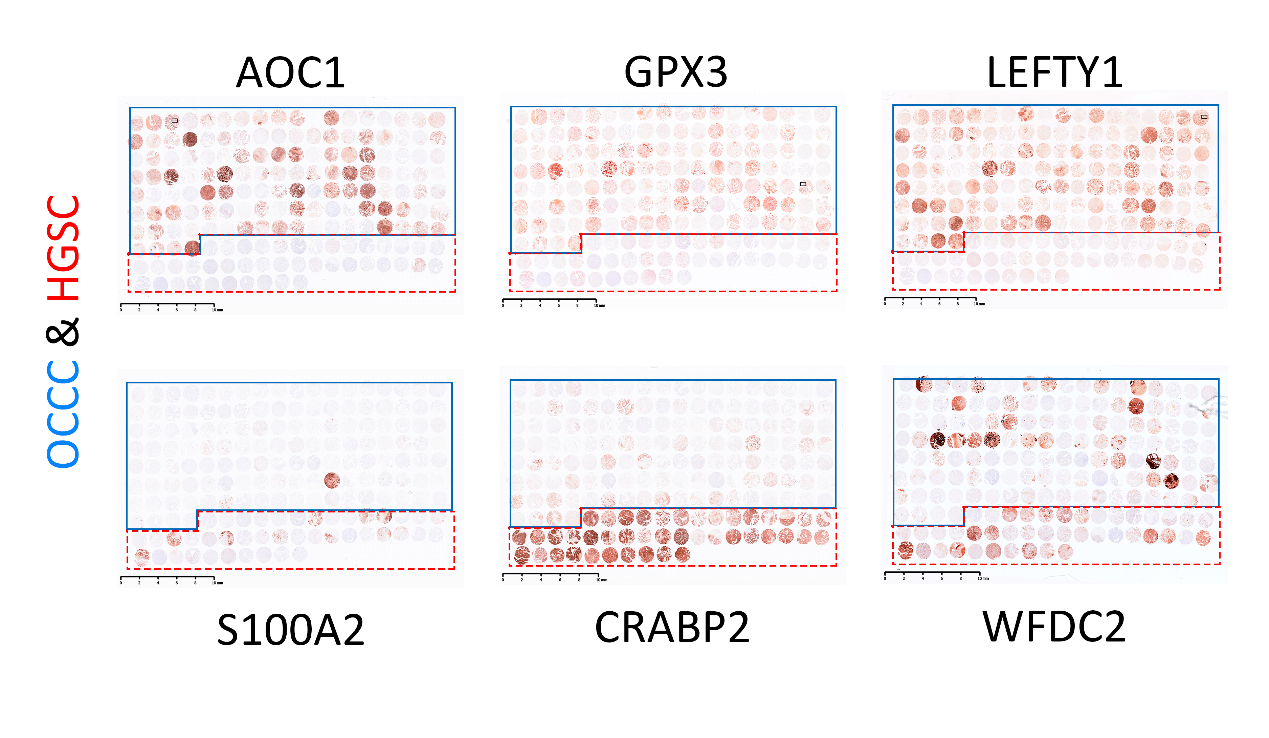


**Fig. S5. Representative images of feature markers in the TMA of OCCC (blue) and HGSC (red) samples.**


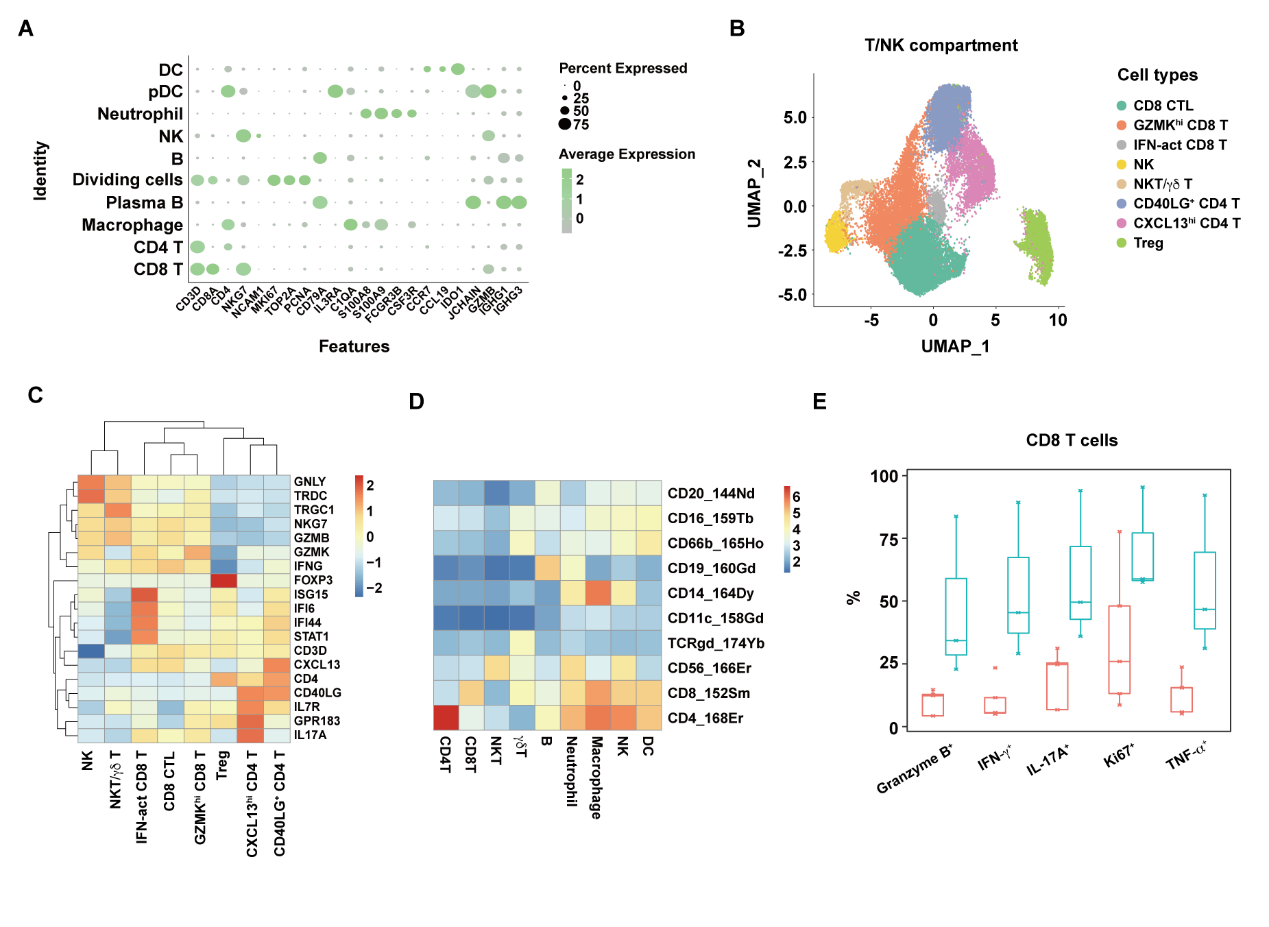


**Fig. S6. The cell composition and immune status in tumors from OCCC and HGSC patients.**

**(A)** Dot plot showing the expression levels of different marker genes in ten clusters obtained from scRNA-seq data. **(B)** UMAP plot showing eight T/NK subpopulations (n = 28396). **(C)** Heatmap showing the average expression of feature markers in different T/NK subsets. (**D)** Heatmap showing the average expression of feature markers in different TIL subsets as determined by mass cytometry analysis. **(E)** Box plots showing the comparison of different marker expression levels in CD8 T cells between the HGSC and OCCC groups. *P* values were calculated by two-sided Wilcoxon test.

**Table S1. Clinicopathological characteristics of patients included in single-cell sequencing**

| Patient | Age | Position | Tumor size (cm) | Histological type | Stage (TNM/FIGO) |
| --- | --- | --- | --- | --- | --- |
| 1 | 64 | left side | 9.5 | OCCC | T1aN0M0/IA |
| 2 | 63 | right side | 8.9 | OCCC | T3cN1aM0/IIIC |
| 3 | 46 | right side | 8.8 | OCCC | T1c3N0M0/IC3 |
| 4 | 34 | right side | 12 | OCCC | T1c1N0M0/IC1 |
| 5 | 51 | right side | 6.9 | OCCC | T1c1N0M0/IC1 |
| 6 | 48 | both sides | 5.5/5.2 | HGSC | T3cN1bM0/IIIC |
| 7 | 49 | right side | 9.3 | HGSC | T1c1N1bM1b/IV |
| 8 | 57 | both sides | 2.6/3.1 | HGSC | T3cN0M0/IIIC |
| 9 | 66 | left side | 6.9 | HGSC | T1c3N0M0/IC3 |
| 10 | 61 | both sides | 2.5/8.6 | HGSC | T3bN0M0/IIIB |

Tumor size was measured on the basis of the longest axis of a primary tumor. The sizes of bilateral tumors were listed as the size of the left tumor/size of the right tumor.

**Table S2. Prognostic value of clinicopathological parameters in OCCC (n=128) and HGSC (n=81) samples based on progression-free survival (PFS)**

|  | OCCC (*p* value) | | HGSC (*p* value) | |
| --- | --- | --- | --- | --- |
|  | univariate | multivariate | univariate | multivariate |
| Age | 0.732 |  | 0.893 |  |
| Stage | 0.010^a^ | 0.014^a^ | 0.911 |  |
| Residual tumor | 0.013^a^ | 0.469 | 0.015^a^ | 0.118 |
| Tumor rupture | 0.372 |  | 0.674 |  |
| Chemoresistance | 0.848 |  | <0.001^a^ | 0.001^a^ |
| AOC1 | 0.056 |  | 0.558 |  |
| GPX3 | 0.021^a^ | 0.029^a^ | 0.835 |  |
| LEFTY1 | 0.585 |  | 0.789 |  |
| S100A2 | 0.489 |  | 0.645 |  |
| CRABP2 | 0.273 |  | 0.680 |  |
| WFDC2 | 0.085 |  | 0.467 |  |

Stages were classified as early stages (FIGO stage I or II) and late stages (FIGO stage III or IV). Chemoresistance was defined as recurrence within six months of completion of platinum-based treatment.

a: statistically significant

**Table S3. Sequences and other detailed information of primers for real-time PCR**

| Gene | Sequence | Expected size of the amplicon (bp) | Annealing temperature (℃) | GenBank Accession number | Primer specificity* |
| --- | --- | --- | --- | --- | --- |
| AOC1 | F: 5′-CCTAAGCAACCAAGAGCTGAA-3′ | 233 | 60 | NM_001091.4 | YES |
|  | R: 5′-CGGTGACATTGGGATGCTCC-3′ |  |  |  |  |
| GPX3 | F: 5′-GGGGATGTCAATGGAGAGAA-3′ | 113 | 60 | NM_002084.5 | YES |
|  | R: 5′-TTCATGGGTTCCCAGAAGAG-3′ |  |  |  |  |
| LEFTY1 | F: 5'-CTGCTGATGGACAAATGCTCTG-3' | 253 | 60 | NM_020997.4 | YES |
|  | R: 5'-ACTTTAGCCCAGATCCAGTGAC-3' |  |  |  |  |
| S100A2 | F: 5′-TGCCAAGAGGGCGACAAGTTCA-3′ | 155 | 60 | NM_005978.4 | YES |
|  | R: 5′-AAGTCCACCTGCTGGTCACTGT-3′ |  |  |  |  |
| CRABP2 | F: 5′-GATGCCTCTTGCAGGGTCTT-3′ | 103 | 60 | NM_001878.4 | YES |
|  | R: 5′-GTGAACCCGGAATGGGTGAT-3′ |  |  |  |  |
| WFDC2 | F: 5′-AGAACTGCACGCAAGAGTG-3′ | 52 | 60 | NM_006103.4 | YES |
|  | R: 5′-TTGAGGTTGTCGGCGCATT-3′ |  |  |  |  |
| GAPDH | F: 5′-TCGGAGTCAACGGATTTGGT-3′ | 181 | 60 | NM_002046.7 | YES |
|  | R: 5′-TTCCCGTTCTCAGCCTTGAC-3′ |  |  |  |  |

The primer specificity was verified by blast (Table S4).

**Table S4.** **Verification of the specificity of primers used in real-time PCR**

| Gene name | Melting curve | Primer blast |
| --- | --- | --- |
| AOC1 |  | 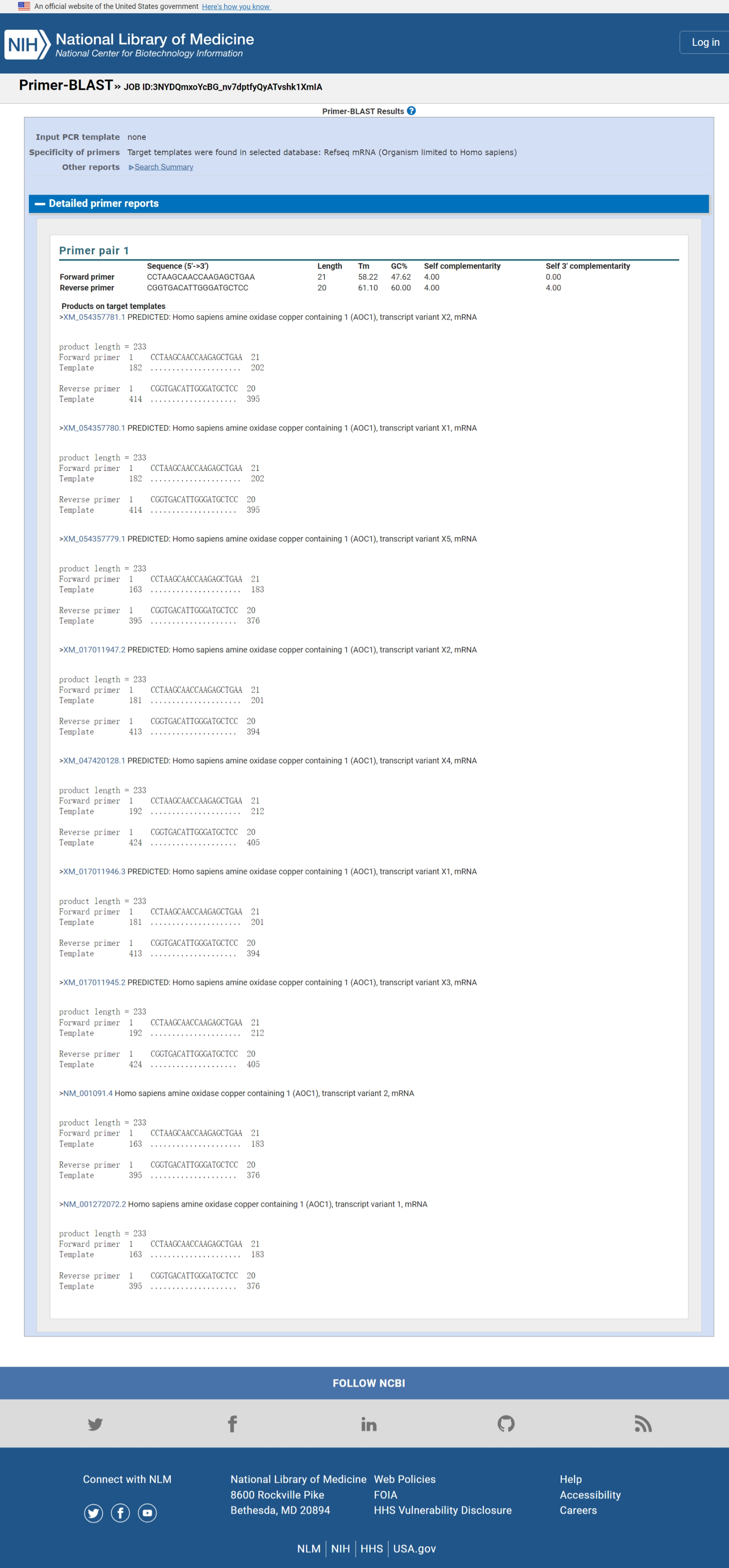 |
| GPX3 |  | 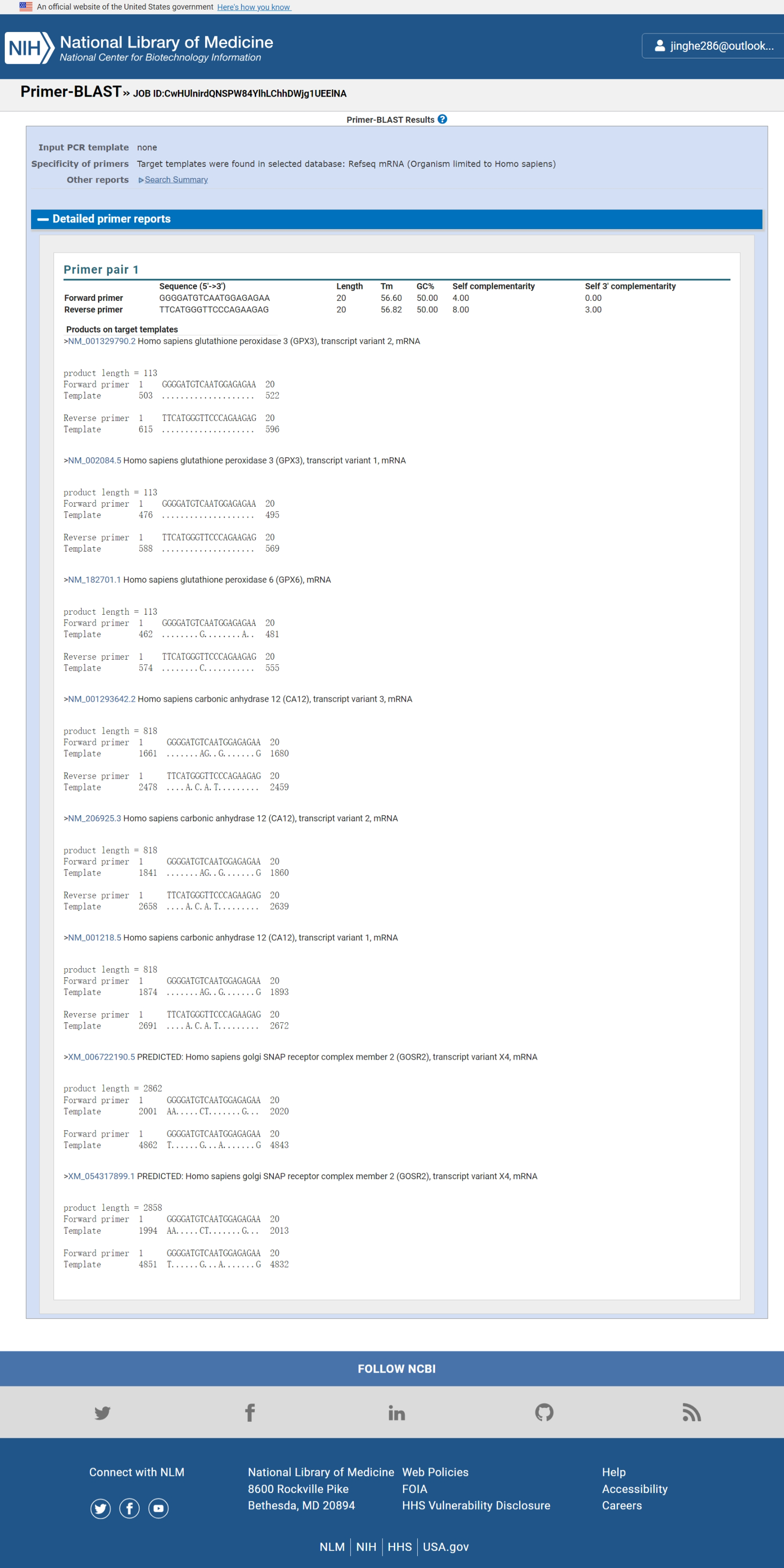 |
| LEFTY1 |  | 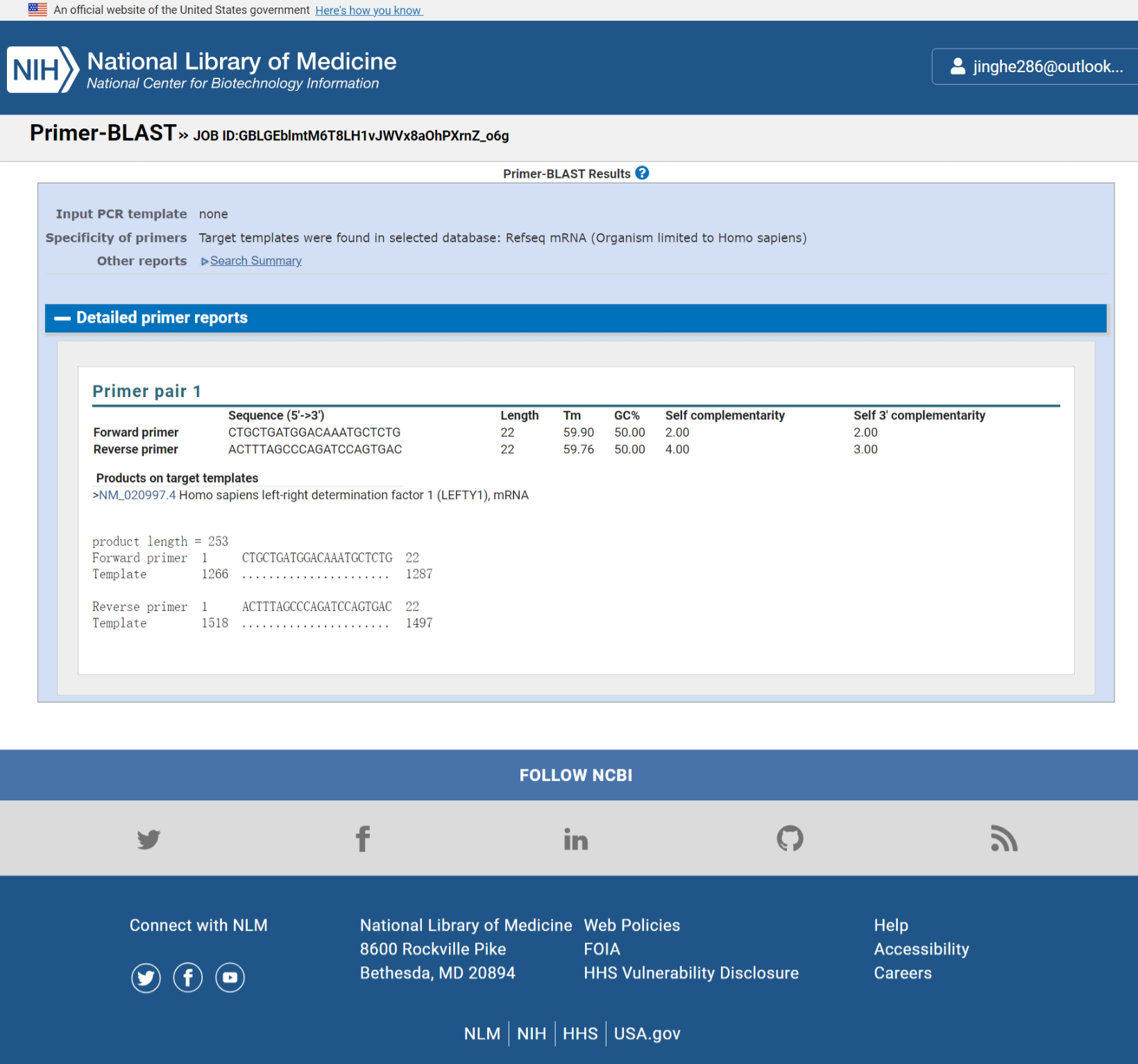 |
| S100A2 |  | 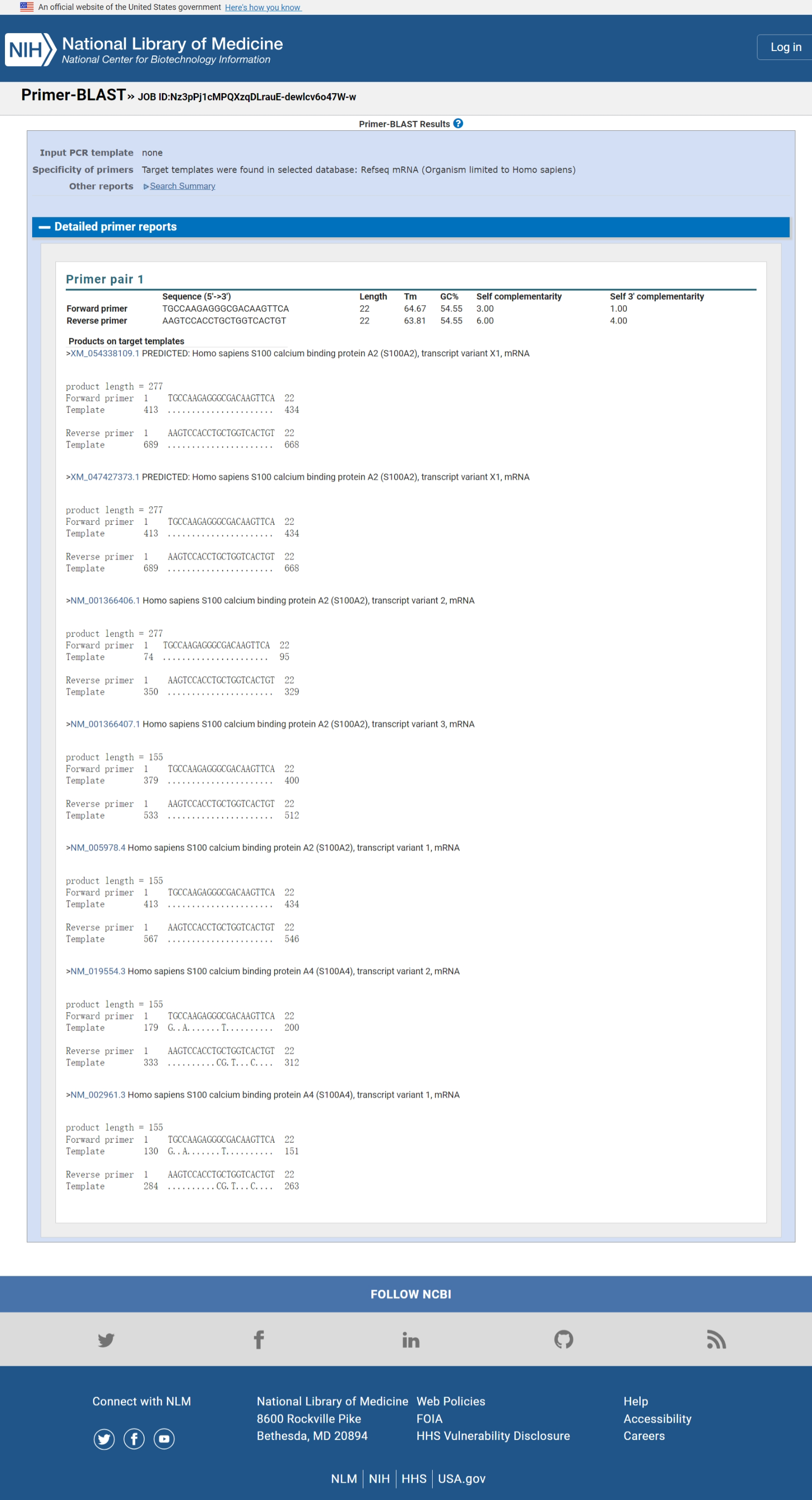 |
| CRABP2 |  | 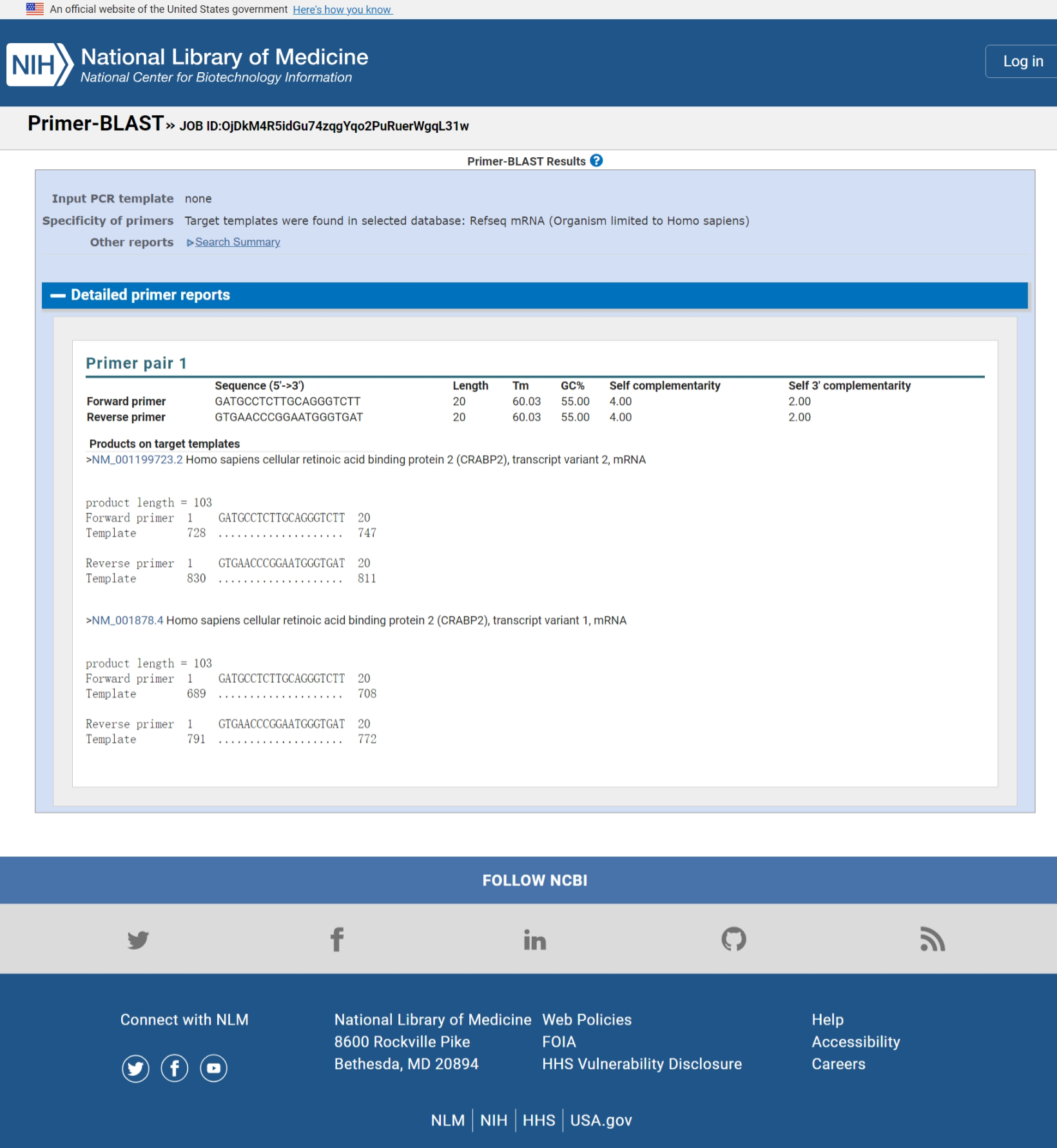 |
| WFDC2 |  | 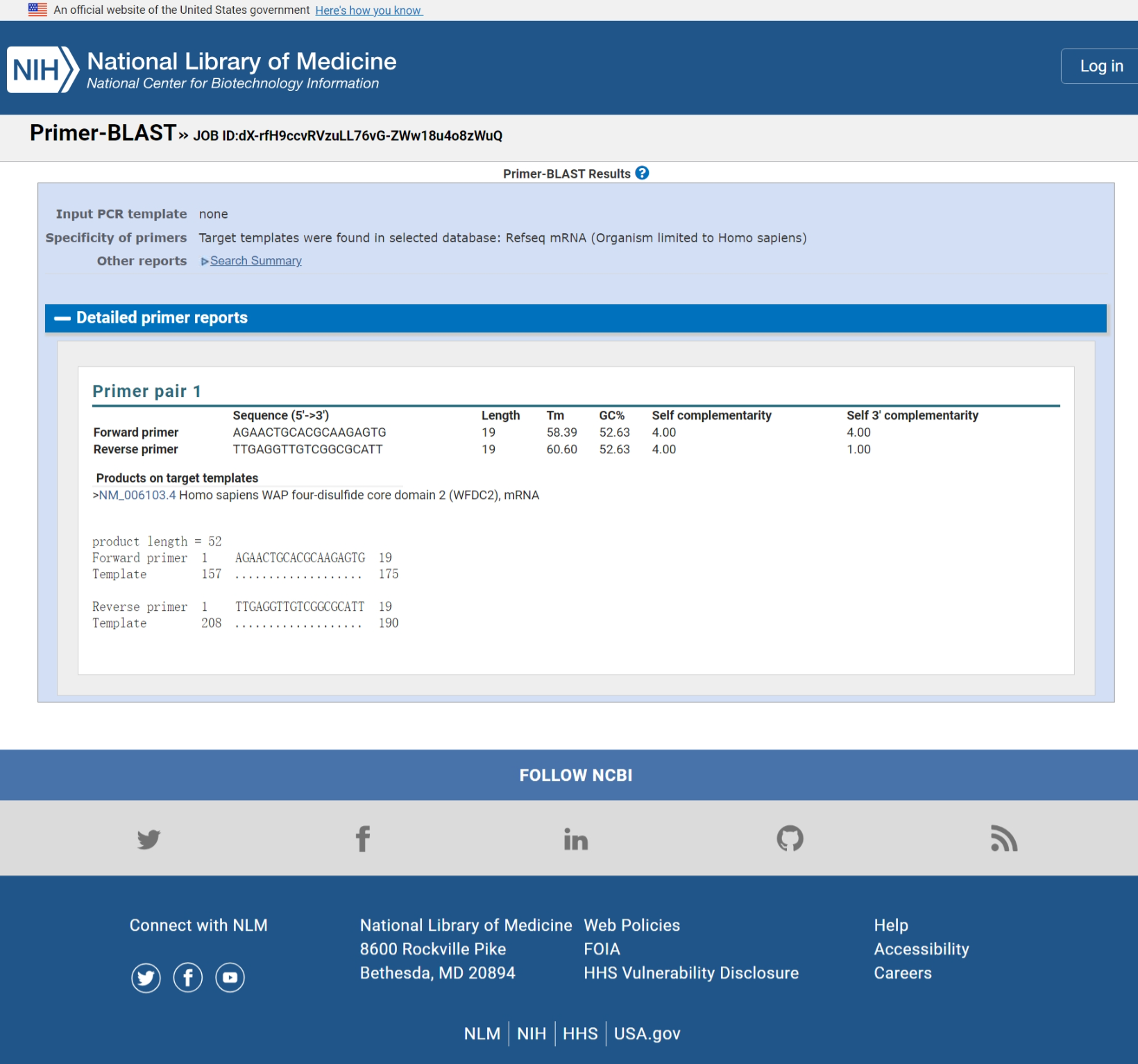 |
| GAPDH |  | 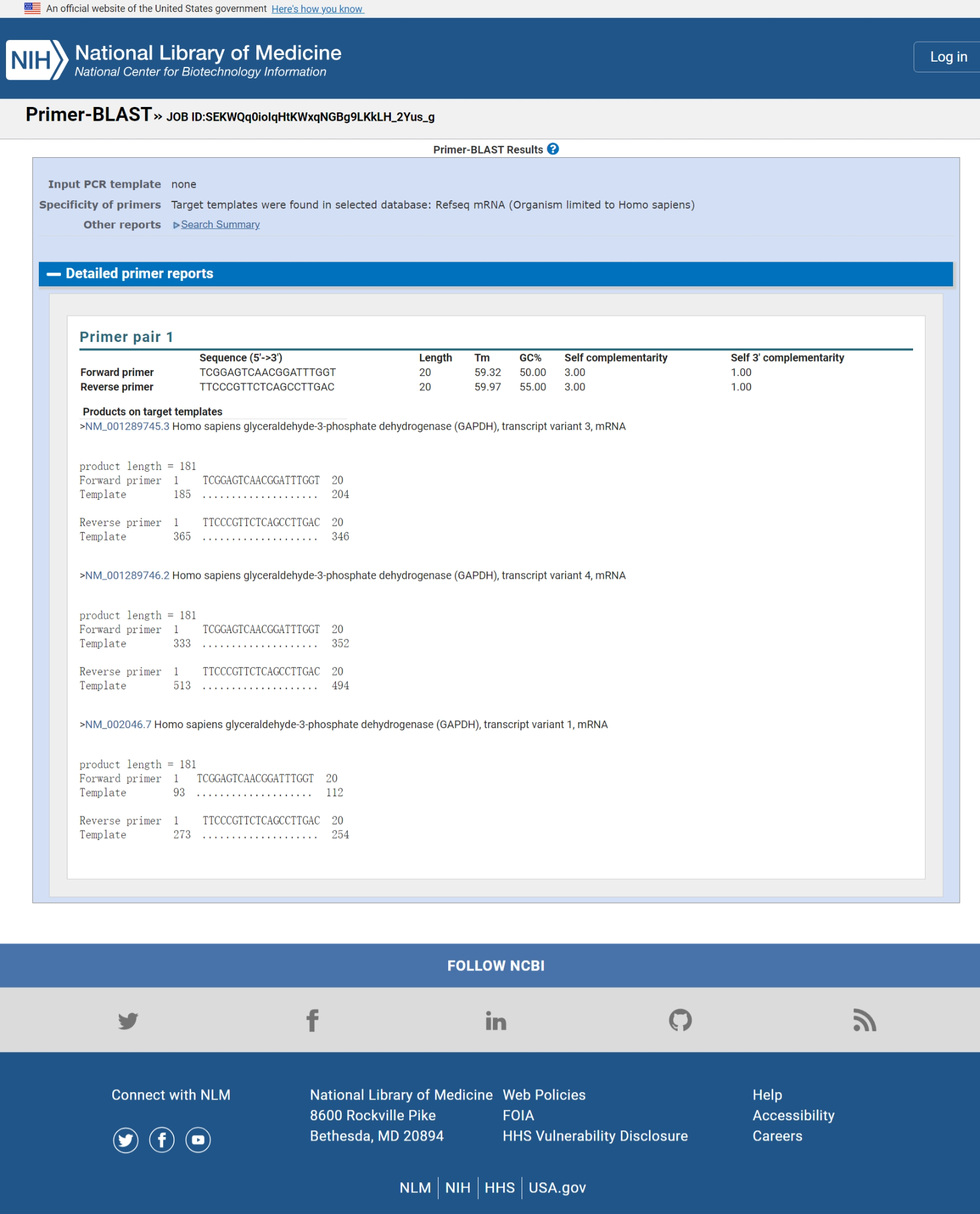 |

**Table S5. Clinical characteristics of the cohort for IHC validation**

|  | OCCC (n=128) | HGSC (n=81) |
| --- | --- | --- |
| Age (mean ± SD) | 51 ± 10 | 57 ± 10 |
| Stage (FIGO) |  |  |
| I/II | 96 (75.0%) | 20 (24.7%) |
| III/IV | 32 (25.0%) | 61 (75.3%) |
| No residual tumor (R0) | 120 (93.8%) | 65 (80.2%) |
| Tumor rupture | 46 (35.9%) | 5 (6.2%) |
| Chemotherapy | 124 (96.9%) | 76 (93.8%) |
| Other therapies | 27 (21.1%) | 41 (50.6%) |
| Chemoresistance | 15 (11.7%) | 2 (2.5%) |
| Recurrence | 23 (18%) | 6 (7.4%) |
| PFS (mean ± SD) | 16 ± 11 | 10 ± 7 |
| Time to recurrence  (mean ± SD) | 12 ± 8 | 13 ± 6 |

Time to recurrence was defined as months between the first surgery and recurrent time, and only patients with recurrence were included.

**Table S6. Primary antibodies utilized in Western blotting and IHC**

|  | Western blotting | IHC |
| --- | --- | --- |
| AOC1 | 1:1000, ab278497, Abcam | 1:2000, ab278497, Abcam |
| GPX3 | 1:1000, ab275965, Abcam | 1:10000, ab200828, Abcam |
| LEFTY1 | 1:1000, #12647, Cell Signaling Technology | 1:5000, ab275965, Abcam |
| S100A2 | 1:1000, ab109494, Abcam | 1:50, #12647, Cell Signaling Technology |
| CRABP2 | 1:1000, 10225-1-AP, Proteintech | 1:500, 10225-1-AP, Proteintech |
| WFDC2 | 1:1000, ab200828, Abcam | 1:500, ab109494, Abcam |

Titer, product number, and company of each primary antibody is included.

**Table S7. The location of expressed protein and the tissue control in IHC staining**

| **Primary antibody** | **Expression** | **Positive control** | **Negative control** |
| --- | --- | --- | --- |
| AOC1 | cytoplasm & cell membrane | kidney | internal control: stromal cells (fibroblast, etc.) |
| GPX3 | cytoplasm | kidney |  |
| LEFTY1 | cytoplasm | pancreas |  |
| S100A2 | cytoplasm & cell nucleus | esophagus |  |
| CRABP2 | cytoplasm & cell nucleus | esophagus |  |
| WFDC2 | cytoplasm & cell membrane | endometrial cancer |  |
